# Evaluation of a Pooled Strategy for High-Throughput Sequencing of Cosmid Clones from Metagenomic Libraries

**DOI:** 10.1371/journal.pone.0098968

**Published:** 2014-06-09

**Authors:** Kathy N. Lam, Michael W. Hall, Katja Engel, Gregory Vey, Jiujun Cheng, Josh D. Neufeld, Trevor C. Charles

**Affiliations:** Department of Biology, University of Waterloo, Waterloo, Ontario, Canada; Institut National de la Recherche Agronomique, France

## Abstract

High-throughput sequencing methods have been instrumental in the growing field of metagenomics, with technological improvements enabling greater throughput at decreased costs. Nonetheless, the economy of high-throughput sequencing cannot be fully leveraged in the subdiscipline of functional metagenomics. In this area of research, environmental DNA is typically cloned to generate large-insert libraries from which individual clones are isolated, based on specific activities of interest. Sequence data are required for complete characterization of such clones, but the sequencing of a large set of clones requires individual barcode-based sample preparation; this can become costly, as the cost of clone barcoding scales linearly with the number of clones processed, and thus sequencing a large number of metagenomic clones often remains cost-prohibitive. We investigated a hybrid Sanger/Illumina pooled sequencing strategy that omits barcoding altogether, and we evaluated this strategy by comparing the pooled sequencing results to reference sequence data obtained from traditional barcode-based sequencing of the same set of clones. Using identity and coverage metrics in our evaluation, we show that pooled sequencing can generate high-quality sequence data, without producing problematic chimeras. Though caveats of a pooled strategy exist and further optimization of the method is required to improve recovery of complete clone sequences and to avoid circumstances that generate unrecoverable clone sequences, our results demonstrate that pooled sequencing represents an effective and low-cost alternative for sequencing large sets of metagenomic clones.

## Introduction

With the advent of high-throughput sequencing, metagenomics has emerged as a powerful way to explore DNA recovered from terrestrial, aquatic and host-associated microbial communities. Sequence-based metagenomics involves bulk sequencing of environmental DNA and has generated a wealth of genome information from myriad environmental samples. With this wealth of sequence data serving as a foundational resource, the stage is set for function-based metagenomics, or functional metagenomics, which is arguably essential for the recovery and annotation of hypothetical proteins with as-yet-unknown functions [1], [2].

Functional metagenomics allows exploration of the densely populated microbial habitats that are rich resources for the discovery of novel enzymes. Applying this approach, the genetic material of the microbial community is extracted from an environmental sample, and the DNA is cloned into appropriate vectors to generate metagenomic libraries that are maintained using *Escherichia coli* as a surrogate host. These libraries may then be subjected to function-based activity screens, either in *E. coli* or various other surrogate hosts, after which positive clones are isolated for analysis.

A critical step in functional metagenomic studies is obtaining DNA sequence for the isolated clones in order to identify the gene(s) responsible for the function(s) of interest, particularly if the goal is to identify novel enzymes. Prior to the existence of high-throughput sequencing, it was, and still is, common to use other methods to identify the gene or operon carried on the insert DNA. One strategy is to Sanger-sequence the clone to obtain a sequence fragment, by primer-walking along the insert [3]–[6] or first subcloning smaller fragments of the insert that carry the activity of interest [7]–[16]. A variant of this strategy is to use transposon mutagenesis, which may be followed by screening for loss of activity [17]–[26]. Regardless of the specific strategy, multiple steps are usually required to obtain sequence data for large-insert clones.

**Figure 1:**
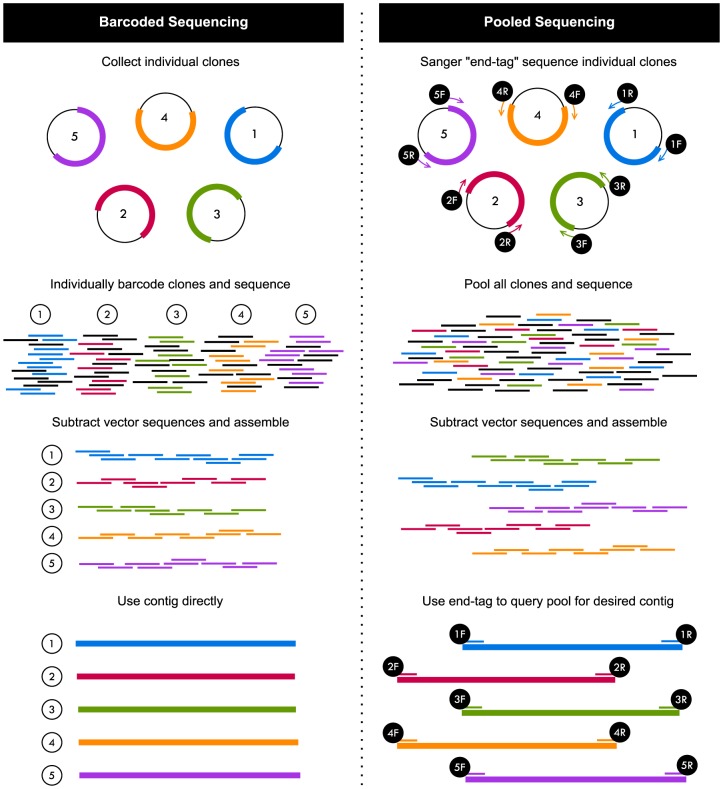
Overview of the two sequencing methods used in this study for sequencing of large-insert cosmid clones. Traditional barcoded sequencing (left) uses DNA barcodes to keep clones as separate samples throughout the sequencing and assembly process. Pooled sequencing (right) involves combining clones into one sample for sequencing and assembly, and subsequently using previously obtained Sanger “end-tags” to retrieve specific clone sequences.

Although current high-throughput sequencing methods are an appropriate scale for sequencing of microbial genomes, the throughput is typically far greater than required for coverage of single clones. This has led to the practice of “multiplexing”, which involves combining multiple clones for sequencing, using DNA barcodes (or indexes) to track sequence reads from individual clones within the larger set (**Figure 1**, Barcoded Sequencing). Examples of this strategy include the sequencing of large-insert clones identified from screens for enzymes involved in dietary fibre catabolism [27], prebiotic breakdown [28], and cellulosic biomass conversion [29]. Barcoded sequencing enables sequence data recovery from many clones simultaneously, yet the cost of barcoding every clone can be several-fold higher than the cost of the sequencing itself. This sample preparation cost can be a bottleneck for the smaller molecular microbiology lab, where isolating clones is relatively easy, but sequence analysis of the clones becomes cost-prohibitive.

We investigated the possibility of circumventing the barcoding step by testing a clone pooling and sequencing approach (**Figure 1**, Pooled Sequencing). As part of this sequencing strategy, end sequences for every clone are generated by Sanger-sequencing; we call these sequences “end-tags” to describe their role in the downstream sequence retrieval process in which we match clones to next-generation sequence data assemblies. In a pooled method, clones are sequenced together and we rely on the post-sequencing assembly process to generate contigs that represent individual clones. After assembly, contigs exist in a pool; to retrieve a specific clone's contig, we use the clone's end-tags to query the pool.

We chose a set of 92 large-insert clones (∼33 kb average insert size, data not shown) that we identified previously from multiple functional screens. We obtained end-tags from Sanger sequencing each clone and, concurrently, we pooled the clones for sequencing and assembly. Though the reduced cost of pooled sequencing is very attractive, we acknowledged that the data obtained could be of poorer quality. While some compromise is of course made in a strategy that seeks economy, we were uncertain about the extent of the trade-off. Therefore, to evaluate the results of the pooled sequencing strategy, we had the same set of 92 large-insert clones sequenced using barcodes, generating sequences to which our pooled sequencing results could be compared. Our aim is not to do a comparison of the two methods to show that the pooled method is superior; rather, our aim is to examine the results from a pooled sequencing approach, using high-quality reference sequences from traditional barcoded sequencing. Although a similar pooled clone sequencing method has recently been described by others for metagenome-derived medium-insert plasmids [30] and large-insert fosmids [31], to our knowledge, we are the first to report the pooled strategy for sequencing of large-insert metagenomic clones while also critically evaluating the performance of this pooled strategy by comparing the results to barcoded reference sequences of the same clones.

## Materials and Methods

### Ethics Statement

Approval for the collection of human fecal samples was obtained from the Office of Research Ethics of the University of Waterloo in Waterloo, Canada, and written consent was obtained from the volunteers. No identification was attached to the collected samples and samples were pooled prior to use.

### Isolation of high molecular weight DNA from environmental samples

Soil samples were obtained from diverse environments across Canada [32]. Information regarding the metagenomic libraries constructed from Canadian soil samples is available online through the Canadian MetaMicrobiome Project website (http://www.cm2bl.org).

Prior to DNA extraction, fecal samples were pre-processed based on the method described by Lee and Hallam [33], by placing 5 g of sample in a mortar with 1 ml of denaturing solution (4 M guanidine isothiocyanate, 10 mM Tris-HCl [pH 8.0], 1 mM EDTA, 0.5% beta-mercaptoethanol). The sample was frozen using liquid nitrogen, ground with a pestle to a homogeneous powder, then transferred to a conical tube for storage at −80°C.

DNA was extracted from soil or feces according to the method described by Zhou *et al.* [34]. Briefly, 5 grams of soil or fecal sample were incubated in 13.5 ml of extraction buffer (100 mM Tris [pH 8.0], 100 mM EDTA, 100 mM sodium phosphate [pH 8.0], 1.5 M NaCl, 1% CTAB), with the addition of proteinase K (to 75 µg/ml), shaking at 37°C for 30 minutes. After adding SDS (to 2% w/v in 15 ml), the sample was incubated at 65°C for 2 h with gentle inversions every 15 minutes. After centrifugation at 6000×g for 10 minutes at room temperature, the supernatant was collected, extracted with chloroform:isoamyl alcohol (24∶1), and DNA was precipitated with 0.6 volumes of isopropanol at room temperature for 1 h. DNA was collected by centrifugation at 6000×g for 20 minutes at room temperature, followed by a 70% ethanol wash. The DNA pellet was suspended overnight at 4°C in 0.5 to 3 ml of either water or TE buffer (10 mM Tris-HCl [pH 8.0] and 0.1 mM EDTA [pH 8.0]).

Extracted DNA was either cloned directly or purified further by synchronous coefficient of drag alteration (SCODA) using the Aurora (Boreal Genomics) according to an established protocol [35]. Crude or SCODA-purified DNA was quantified by gel electrophoresis, using bacteriophage lambda DNA as a standard.

### Isolation of high molecular weight DNA from pure cultures

DNA was isolated from liquid bacterial cultures based on a method described by Charles *et al.* [36]. Briefly, cells were cultured in 50 ml of liquid media, and the cell pellets were recovered after centrifugation at 7000×g for 5 minutes at room temperature. Cells were washed with 8 ml of wash buffer (10 mM Tris [pH 8.0], 25 mM EDTA [pH 8.0], 150 mM NaCl), and resuspended in 4 ml of buffer (10 mM Tris [pH 8.0], 25 mM EDTA). The following were added, to a final volume of 5 ml: NaCl (to 0.5 M), proteinase K (to 0.5 mg/ml), and lysozyme (to 2.5 mg/ml). After incubation at 37°C for 30 minutes with shaking, 250 µl of 20% SDS were added, the mixture was incubated at 65°C for 60 minutes, then centrifuged at 6,000×g for 10 minutes at room temperature. The supernatant was collected, and DNA precipitated with 0.5 volumes of 7.5 M sodium acetate on ice for 10 minutes. The mixture was centrifuged at 10,000×g for 15 minutes, the supernatant was collected and centrifuged at 8,500×g for 10 minutes to further clear the supernatant. The supernatant was collected and DNA was precipitated with 1 volume of isopropanol at room temperature for 30 minutes. DNA was spooled out, dipped in a 70% ethanol wash, and placed in a microfuge tube. The tube was centrifuged at 16,000×g for 1 minute, the supernatant was removed, and the pellet was allowed to dry. Finally, the pellet was allowed to dissolve in 2 ml of TE. The DNA was quantified by gel electrophoresis, using bacteriophage lambda DNA as a standard.

### Construction of large-insert metagenomic cosmid libraries

The cosmid vector pJC8 (Genbank accession KC149513) formed the backbone of all metagenomic libraries constructed in this study. In addition to constructing new libraries, existing metagenomic clones were used from previous libraries [37], constructed in the cosmid vector pRK7813 (Genbank accession KC442292; [38]). All libraries have entries in the NCBI BioSample database [39], and details regarding the libraries used in this study are summarized in **Table 1**.

**Table 1:** Metagenomic and genomic libraries used in this study.

Internal Library ID	NCBI BioSample ID	DNA source	Approximate no. of clones	Vector backbone	Reference
12AC*	SAMN02324088	soil (agricultural)	80,000	pJC8	[40]
BF1	SAMN02324093	*Bacteroides fragilis* NCTC 9343	18,000	pJC8	this study
BT1	SAMN02324089	*Bacteroides thetaiotaomicron* VPI 5482	8,000	pJC8	this study
CLGM1	SAMN02324081	human feces	42,000	pJC8	this study
CX3	SAMN02324235	activated sludge (pulp and paper)	2,500	pRK7813	[37]
CX4	SAMN02393652	activated sludge (pulp and paper)	3,900	pRK7813	[37]
CX6	SAMN02393657	activated sludge (municipal)	3,300	pRK7813	[37]
CX9	SAMN02393684	soil (creek)	22,000	pRK7813	[37]
CX10	SAMN02393686	soil (creek)	8,700	pRK7813	[37]

*from the Canadian MetaMicroBiome Library collection, http://www.cm2bl.org.

Libraries were constructed as previously described [40]. Briefly, the vector pJC8 was digested with *Eco*72I/*Pml*I to produce blunt ends and then dephosphorylated. The backbone was purified from the 0.8 kb gentamicin resistance gene stuffer, either with an EZ-10 Spin Column DNA Gel Extraction Kit (BioBasic) or by electroelution. The high-molecular-weight DNA extracted from either environmental samples or pure culture (up to 25 µg of either crude or purified DNA) was size-selected by pulsed-field gel electrophoresis (PFGE) using a CHEF MAPPER Pulsed Field Gel Electrophoresis System (Bio-Rad). The gel fragment containing DNA of approximately 40–70 kb was excised, then electroeluted and concentrated using an Amicon Ultra Centrifugal Filter with 30 kDa MWCO (Millipore). Purified DNA (2.5 µg) was end-repaired using the End-It DNA End-Repair Kit (EpiCentre). A phenol:chloroform extraction was performed to remove T4 polynucleotide kinase, and DNA was precipitated, resuspended in TE, and quantified by gel electrophoresis, using bacteriophage lambda DNA as a standard. The purified and blunt-ended DNA was then ligated to the linearized cosmid vector. Ligations were carried out at 14°C overnight with Fast-Link DNA Ligase (EpiCentre), using 500 ng of end-repaired insert DNA and a vector-to-insert molar ratio of 10∶1. Ligations were packaged into lambda phage heads using Gigapack III XL Packaging Extract (Stratagene) according to the manufacturer's instructions, and the final phage suspension was stored at 4°C.

To prepare cells for transduction, *E. coli* HB101 was streaked from frozen stock onto LB agar, and a single colony was then inoculated into 5 ml of LB. The culture was grown overnight at 37°C, was used to inoculate 5 ml of LB supplemented with 0.2% maltose and 10 mM MgSO_4_. The culture was grown to an OD_600_ 0.8 (Spectronic Spec 20D). Cells were pelleted by centrifugation, resuspended in 2.5 ml of LB supplemented with 10 mM MgSO_4_, and held on ice. For an estimate of phage concentration, 10 µl phage were mixed with 90 µl of cells, and the mixture was incubated at room temperature for 30 minutes, and moved to 37°C for 30 minutes. Cells were pelleted by centrifugation and plated on LB with 20 µg/ml tetracycline to select for transductants. Plates were incubated overnight at 37°C, and colonies were counted to estimate phage concentration in the suspension. Finally, the transduction was scaled up to achieve approximately 1000 colonies per plate. Several plates were counted for an estimate of metagenomic library size, and then pooled and stored at −80°C. For regular use, libraries were propagated from the original frozen stock. For an estimate of average insert size, library stocks were streaked onto LB with 20 µg/ml tetracycline, and colonies were selected at random for restriction analysis.

### Functional screens and positive clones

Various function-based screens were performed in our laboratory, including screens for antibiotic resistance genes, conjugation genes, and carbohydrate utilization genes. Tens to hundreds of positive clones were isolated from each screen although 92 distinct clones (based on restriction enzyme digestion patterns) were chosen for full sequencing. The list of clones and the screens from which they were isolated are provided (**Table S1**). Cosmid clone DNA was isolated from either *E. coli* HB101 or DH5á.

### Barcoded sequencing

Cosmid DNA was prepared from *E. coli* DH5á using a GeneJET Plasmid Miniprep Kit (Thermo Scientific), and 1–2 µg of DNA from each of the 92 samples was adjusted to >25 ng/µl. Samples were submitted to the BC Cancer Agency at the Michael Smith Genome Sciences Centre for individual barcoding and 75-base paired-end sequencing on the Illumina HiSeq 2000 platform, using in-house protocols and reagents for library construction. Clones were sequenced to a read depth of approximately 9000-fold, on average (**Table S9** and **Figure S2**). This high coverage was ideal for a high-quality reference data set. Vector sequences were subtracted from the raw data by comparing all reads against the vector backbone using BLAST (with a requirement for 100% identity), and the data were assembled using ABySS version 1.3.2 [41]; default settings were used, with the exception of a k-mer length of 64. At the time of assembly, the complete sequence of the cosmid vector pJC8 was not yet available; as a result, vector subtraction used the closely related parent vector pRK404 (Genbank accessionAY204475; [42]), and assemblies were checked subsequently for remaining vector sequences.

After assembly, the barcoded sequencing data were prepared in order to use as a reference for evaluation of the pooled sequencing data. For the majority of clones, assembly resulted in a single contig, usually exceeding 30 kb, as expected. For cases in which assembly resulted in more than one contig, contigs were manually checked for sequences from contaminating *E. coli* genomic DNA, helper plasmids, and cloning vectors, and those contigs were removed. For 3 clones, multiple contigs remained, indicating the samples may have been insufficiently sequenced, resulting in gaps. Accordingly, we concatenated the multiple large contigs and treated them as one contig. Using the described strategy, reference contigs were obtained for 77 out of 92 clones. The average contig length was 33.5 kb, with the largest being 47.2 kb and the smallest 1.8 kb. Though our cloning strategy enriches for high-insert clones, we have occasionally observed smaller inserts after carrying out functional screening. These smaller inserts may have arisen from recombination and subsequent loss of cloned DNA after the library construction process. Sequence data have been made available for download (see below). Barcodes are provided in **Table S2**.

### Sanger end-sequencing and pooled sequencing

Cosmid DNA was prepared from *E. coli* DH5á using a GeneJET Plasmid Miniprep Kit (Thermo Scientific). Aliquots of 100 ng from each of the 92 samples were pooled and concentrated to 125 ng/µl. The pooled samples were sequenced by the Beijing Genomics Institute (BGI) using 90-base paired-end sequencing on the Illumina HiSeq 2000 platform, using in-house protocols and reagents for library construction. Clones were sequenced to a read depth of approximately 900-fold on average (**Table S9** and **Figure S2**), upon recommendation of >100-fold coverage. The service provider subtracted vector sequences using SOAPaligner version 2.21 [43] (again, using pRK404), and completed assembly using SOAPdenovo version 1.05 [44], using a k-mer size of 31, and BWA version 0.5.8 [45]. This resulted in 563 contigs ranging between 0.5 kb to 97.7 kb, with a mean contig length of 11.7 kb. Contigs exceeding the expected insert size were determined to be *E. coli* genomic DNA contamination, the presence of which did not interfere with clone sequence retrieval as retrieval is done using clone end sequences.

Concurrent to pooled sequencing, samples were end-sequenced by Sanger sequencing at BioBasic Inc., Lucigen Corporation, or The Centre for Applied Genomics, to generate end-tags. One or both end sequences were obtained for 83 out of 92 clones (**Table S3**). Sequencing primers used were standard M13 forward and M13 reverse from the sequencing facility, or custom primers JC102 (5′TAACAATTTCACACAGGAAACAGCTATGAC) and JC103 (5′GCGATTAAGTTGGGTAACGCCAGGGTTTTC). The obtained end-tags were then used to query the pooled sequencing results, using NCBI nucleotide BLAST [46] running the Megablast algorithm. In this manner, contigs were retrieved from the pool for each clone (**Table S4**). Pooled sequence data and end sequence data have been made available for download (see below).

### *E. coli* genomic DNA contamination analysis

Because contamination of samples with *E. coli* genomic DNA was found to affect downstream assembly of barcoded samples, raw data were used to estimate percent contamination. The genome of *E. coli* DH1 (Genbank accession CP001637) was used as a reference, being the parent of DH5á, the strain used in the lab for cosmid propagation. All sequence reads were examined for similarity to the DH1 genome, using a criterion of 100% identity. Contamination ranged from 1% to approximately 50% in the barcoded samples (**Figure S1**) and 5% in the pooled sample (data not shown).

### Read depth analysis

Read depth was estimated for each clone, for both barcoded sequencing and pooled sequencing. In both cases, the barcoded clone sequence was used as the reference sequence; raw reads were aligned to the reference sequence using BWA version 0.7.6a [45] and depth at each base was counted using SAMtools version 0.1.18 [47]. Average read depth for each clone is provided (**Table S9**) as well as read depth at every base across each clone (**File S1**).

### Clone sequence similarity analysis

Sequence similarity was estimated for all clones using BLAST [46] on the barcoded reference sequences, specifically blastn with an e-value cut-off of 0.001. In each pair-wise comparison, the total alignment length was divided by the shorter clone length to obtain a similarity value between 0 and 1. Clones with no sequence similarity identifiable by BLAST were assigned a similarity value of 0.

### Data

Raw sequence data are available at the NCBI Sequence Read Archive under Study SRP031898. Accession numbers for all SRA Experiments are provided (**Table S5**) as are Sanger end sequences for the pooled sequencing strategy (**Table S3**) and barcode information for the barcoded sequencing strategy (**Table S2**). In addition, raw data and relevant information for both barcoded and pooled sequencing may be accessed at http://www.cm2bl.org/~data

## Results and Discussion

### Pooled and barcoded sequencing results

We evaluated data obtained from a pooled sequencing strategy, a more economical approach than traditional barcode-based sequencing. To do this, a total of 92 cosmid clones were subjected to both pooled sequencing and barcoded sequencing. As a result of using different providers, we obtained unequal coverage between the two sequencing approaches (**Figure S2**); however, it was the barcoded strategy that had the greater coverage, which was ideal for its use as the reference data set.

Of the 92 large-insert cosmid clones, 19 were excluded from subsequent analyses due to incomplete sequencing data. Of the excluded clones, 15 clones had insufficient barcoded sequence data for successful assembly (as described in **Methods**). We found that these samples had high contamination of *E. coli* genomic DNA and/or mobilizer plasmid DNA. To examine the effect of contamination on clone assembly, we estimated percent *E. coli* contamination in each of the 92 samples (see **Methods**); we found that contamination ranged from 1% to nearly 50%, and, not surprisingly, that the higher the contamination, the less likely a successful assembly (**Figure S1**). The remaining 4 of the 19 clones repeatedly failed Sanger end sequencing reactions, possibly due to secondary structure associated with the insert DNA. In our experience, it is occasionally difficult to obtain Sanger reads for certain clones, which we speculate may be caused by such secondary structure effects.

In total, 73 clones yielded sufficient data for evaluation of the pooled sequencing results, using the barcoded sequencing results as a reference.

### Evaluation of pooled sequencing results

Using the set of 73 clones, we evaluated the accuracy and completeness of the pooled sequencing approach (retrieved contigs for all clones are given in **Table S4**). For each clone, we used the barcoded sequencing result (i.e., the “barcoded contig”) as the reference to which we compared the pooled sequencing result (i.e., the retrieved “pooled contig”). Specifically, the retrieved pooled contig was aligned to its respective barcoded contig, using NCBI nucleotide BLAST [46] running the Megablast algorithm. By aligning the pooled contig to the barcoded contig for each clone, we were able to quantitatively assess our pooled sequencing approach, by obtaining values for percent identity (i.e., did pooled sequencing return the expected sequence for the clone?) and percent coverage (i.e., did pooled sequencing return the expected length for the clone?).

**Figure 2:**
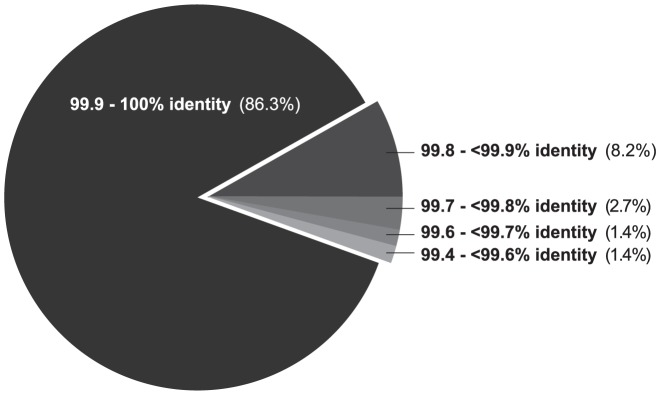
Alignment identity between pooled sequencing result and barcoded sequencing result. For all 73 clones, end-tags were used to retrieve contigs from pooled sequencing results; retrieved contigs were aligned to the reference barcoded sequencing result, and clones were binned by percent identity.

Our initial reservations about a pooled sequencing strategy centred on one major issue, which was that assembly of reads generated from a pooled sample may result in chimeric assemblies – that is, assemblies that are derived from more than one clone. When we aligned retrieved pooled contigs to barcoded contigs for each clone, we found that the majority of clones showed alignments of greater than 99.9% identity, with identity values ranging from 99.4–100.0% (**Figure 2**). Identity values showed high accuracy and little variability, indicating that the pooled sequencing strategy is capable of generating consistently accurate sequence data. Contrary to our concerns, the alignments showed that we did not encounter problems with chimeric sequences, and that most sequences had an error rate of less than one base per thousand. Indeed, this might be an overestimation of the error because the pooled sequencing and assembly method may mask the presence of single nucleotide polymorphisms (discussed below).

**Figure 3:**
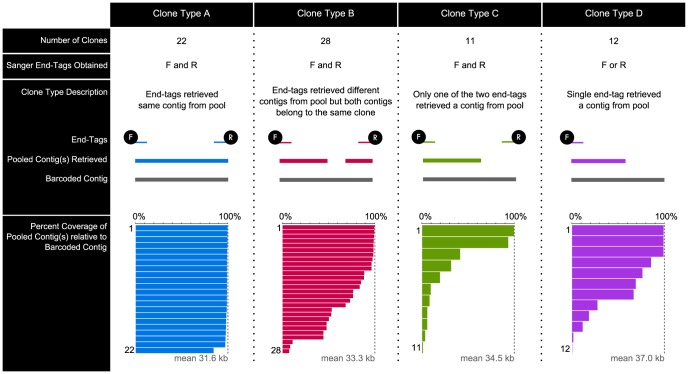
Percent coverage of pooled sequencing result relative to barcoded sequencing result. Each of the 73 clones was categorized into Clone Types A, B, C, or D by the number of end-tags obtained (one or two), whether the end-tag retrieved a contig from the pool, and the completeness of the retrieved pooled sequencing result relative to the reference barcoded sequencing result (full or partial coverage). Clone Type descriptions are given above.

We used the same alignments to determine clone coverage obtained by the pooled method and, in contrast to identity, we found that sequence coverage of pooled clones varied widely. To assess clone coverage, we first categorized the 73 clones into Clone Types (Type A, B, C, or D) based on whether we obtained one or both end-tags, whether the end-tags were able to retrieve a pooled contig, and whether one or two pool contigs were retrieved (**Figure 3**; designations for each clone are given in **Table S6**). Type A represents the ideal outcome, in which the two end-tags retrieved the same contig from the pool; in this case, pooled sequencing resulted in ∼100% coverage for the clone. Type B represents a scenario in which end-tags retrieved different contigs due to a gap in coverage in the middle of the clone. Types C and D represent cases in which coverage was variable and likely underestimated, given that one of the two end-tags was either missing or failed to retrieve a contig. Coverage was highly variable, ranging from 0.4–100.0% over the 73 clones analyzed (**Figure 3**; percent coverage for all clones is given in **Table S7**).

**Figure 4:**
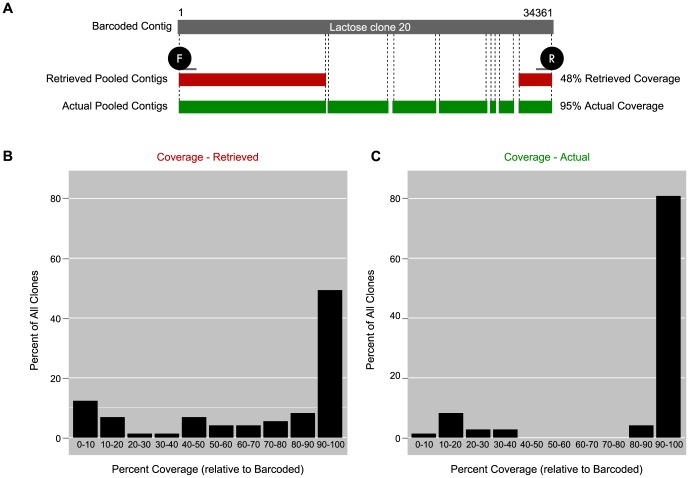
Retrieved coverage and estimated actual coverage of pooled sequencing relative to barcoded sequencing. (A) An example clone, Lactose clone 20, shows retrieved coverage at 48% (using end-tags as queries), but an actual coverage of 98% (using barcoded result as query). (B and C) Percent coverage for each of the 73 clones, binned in ten-percent increments. Retrieved coverage (B) is compared to estimated actual coverage (C).

To determine how well the pooled sequencing strategy worked overall, we used the same coverage data (from **Figure 3**) to bin the 73 clones by coverage (**Figure 4B**). About one-half of the clones showed a retrieved coverage of 90–100%, with an overall average coverage of 71%. We next asked whether the retrieved coverage was an underestimation of the actual coverage achieved by pooled sequencing. To obtain an estimate of the actual coverage, we accounted for unretrieved clone sequences in the pooled sequencing results, which would have occurred due to sequencing gaps, resulting in multiple contigs for a single clone. We thought to compare the retrieved coverage to the actual coverage because the comparison may help to determine whether increasing sequencing depth could increase clone coverage. To recover unretrieved sequences for a clone, we used the reference barcoded sequencing result to query the pool (rather than using the end-tags). When we used the specific end-tags for Lactose clone 20 to retrieve its sequence from the pool, we obtained a retrieved coverage of 48% (**Figure 4A**); however, when we used the reference barcoded sequencing result to query the pooled sequencing results, the coverage improved to 95%. This latter value reflects the actual sequence coverage of the clone found in the pooled sequencing results. We used this strategy to correct for unretrieved sequences for all 73 clones, using a 250-base length cut-off and 99.6% identity cut-off; after this correction, coverage improved to an average of 85%, with over 80% of the clones showing 90–100% coverage (**Figure 4C**).

These data suggest that an increase in the sequencing depth of the pooled strategy may help to increase clone coverage, as this should reduce the occurrence of gaps that prevent retrieval of the full clone sequence. Indeed, others have shown full recovery of circular DNA molecules using a pooled sequencing approach in other applications. For example, bulk sequencing of the plasmid fraction of an activated sludge metagenome resulted in the complete assembly of forty plasmids, which were confirmed to be closed circular replicons by PCR [48], and pooled sequencing of mitochondrial genomes resulted in complete assembly of each, although the authors found that *de novo* transcriptome assemblers, designed for handling reads with differential coverage, provided much better assembly then assemblers meant for genomes [49]. Together, these results support our findings that a pooled strategy can be an effective alternative.

### Clones with sequence similarity may have poor recovery in pooled sequencing

**Figure 5:**
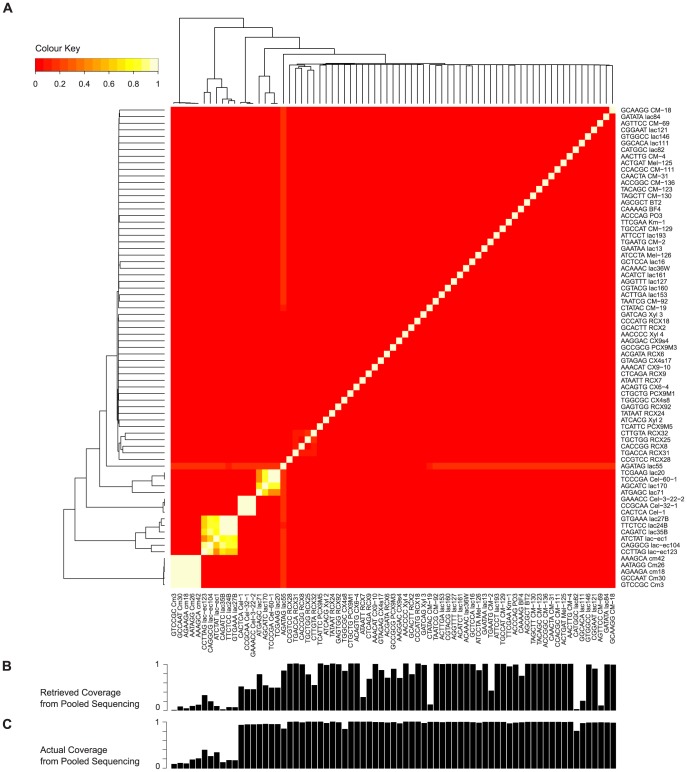
Heat map of clone sequence similarity and corresponding bar plots of clone coverage. Pair-wise sequence similarity is shown for all 73 clones (A), juxtaposed to their pooled sequencing coverage, showing both retrieved coverage (B) and actual coverage (C).

To determine if factors other than depth of sequencing affect clone coverage, we examined the read depth of each clone as well as the sequence similarity between clones. To do the latter, we performed an all-by-all pair-wise BLAST comparison of clones, using their barcoded reference sequences (see **Methods**). We found that the majority of the 73 clones had little or no sequence similarity to any other clone in the pool (**Figure 5A**). However, some clones did have sequence similarity; furthermore, the clones that had sequence similarity were often the same clones that had poor retrieved coverage from pooled sequencing (**Figure 5B**). This was particularly striking when looking at the actual coverage (**Figure 5C**), suggesting that increasing the depth of sequencing may improve clone coverage from pooled sequencing, but only for those clones that do not have sequence similarity to other clones present in the pool.

This reasoning was further corroborated by read depth analysis of each of the 73 clones. To examine the relationship between read depth and pooled sequencing coverage, we aligned raw pooled sequencing reads to the barcoded reference sequence for each clone (see **Methods**), and plotted the read depth against both the retrieved and actual coverage (**Figure S3**). We found that for a number of clones, the read depth was particularly high and yet the coverage was unusually low; upon inspecting the identity of these clones, we found them to be the same clones that shared sequence similarity. Perhaps not unexpectedly, our results suggest that when clones have sequence similarity, pooling and fragmenting the DNA for sequencing causes: (a) an overrepresentation of similar sequences in the pooled sequencing data, and (b) difficulty in assembling the sequences, leading to lack of coverage for the clones from which the sequences originate. There may be other factors that impact the success of pooled sequencing and assembly, such as the presence of repetitive sequences, but our results suggest that sequencing depth and clone sequence similarity are two significant factors.

### Consensus assemblies: a caveat of the pooled approach

**Figure 6:**
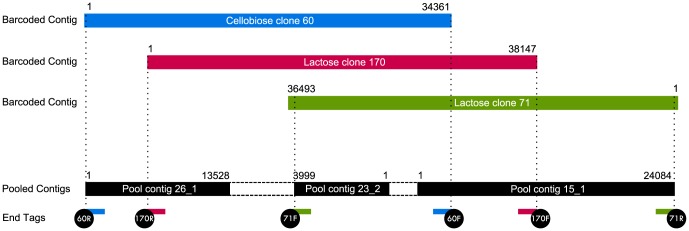
Overlapping clones assemble into one contig. Three overlapping clones as revealed by barcoded sequencing (above) and pooled sequencing (below). Locations of end-tags are indicated by vertical dashed lines. White dashed boxes indicate gaps in the pooled sequencing data; black boxes indicate a contig. Lengths of all contigs are given.

Due to the nature of the pooled assembly, overlapping clones assemble into larger contigs. Indeed, three clones were determined to be overlapping by the barcoded sequence data, as well as the pooled sequence data (**Figure 6**). In the latter, three contigs were retrieved from the pool using their six end-tags; more than one contig was retrieved due to incomplete sequencing and/or assembly by the pooled method, as discussed above (i.e., **Figure 4** and **Figure 5**). Although this larger contig is derived from three clones, we maintain that such a contig should not be classified as chimeric because it represents the metagenomic DNA as it would be found in nature. Furthermore, individual clone sequences can be easily delineated from the greater contig by alignment of clone end-tags to the contig (as illustrated in **Figure 6**).

We view this particular caveat of pooled sequencing as a positive aspect rather than a negative one, because clones from different screens can be immediately identified as overlapping simply from the clone sequence retrieval process. That being said, the assembly of a consensus sequence from overlapping clones may imply a loss of clone-specific information. It is possible that, in some cases, overlapping clones represent different strains of the same microorganism, or different alleles of the same genes(s). Through pooled assembly and depending on the assembler parameters, such clone-specific allelic information, in the form of single nucleotide polymorophisms (SNPs) or similar small sequence variations, may be lost – that is, the final consensus sequence may represent only the most frequent allele. If it should arise, the issue of information loss for allelic variations may be remedied by further analysis. For example, if clones were determined to be overlapping from the consensus contig obtained from pooled sequencing, it would be possible to examine the raw reads to determine if SNPs are present. If so, sequencing primers could be designed for the target loci to determine exactly which SNP(s) belong to which clones in the physical DNA collection.

### Improvements and considerations

In this study, we investigated the quality of data obtained from pooled sequencing because this strategy offered an economical solution to the high cost of traditional barcoded sequencing. At the time this work began, there was a large cost difference in the two services that were available (**Table S8**). Since then, this difference has decreased, and it is likely that it will continue to do so with further developments in sequencing technology. At least for the time being, however, pooled sequencing remains a more affordable option for functional metagenomics research, particularly if a large number of clones must be sequenced.

In our workflow, we concurrently had clones analyzed by pooled sequencing and by Sanger sequencing (for the generation of end-tags); we did this concurrently because we anticipated a lengthy turnaround time for the Illumina sequencing results, which is typically (and was in fact) the case. However, given our experience, we recommend obtaining end sequences for all clones before carrying out pooled sequencing, due to the difficulty of Sanger-sequencing certain clones. Without two end-tags for each clone, it becomes difficult to retrieve the corresponding contig from the pool without further work, such as subcloning and sequencing fragments of the insert (which would negate the ease and economy of the pooled sequencing strategy).

Assembly for both the barcoded and the pooled sequencing strategies revealed contamination with *E. coli* genomic DNA sequences, indicating that minipreps of cosmid clones contained host DNA. Similar results were reported for genomic library BAC clones isolated for pooled sequencing [50]. Such contamination adds undesired DNA template to the sequencing reaction, affecting required-depth-of-coverage calculations, and possibly leading to insufficient sequencing and poor clone sequence recovery. This may have been a problem in our own incomplete recovery for the pooled strategy. We recommend removing contaminating genomic DNA by pre-treatment of samples with Plasmid-Safe DNase (EpiCentre), which may help reduce genomic contamination up to ten-fold [51]. Clone sequence recovery was not problematic in the barcoded sequencing strategy because the sequencing depth was extremely high for the purpose of generating high-quality reference sequence data (see **Methods**).

Another consideration for pooled sequencing relates to the problem of sequence similarity (**Figure 5**). Our results indicate that clones that have sequence similarity are problematic in a pooled strategy, likely due to difficulties in assembling the similar reads and resulting in poor clone sequence recovery. The simple solution would be to avoid pooling clones that share sequence similarity, but this remains a difficult, if not impossible, task without prior knowledge of the clone sequence. A possible way to reduce the potential for sequence similarity may be to assemble pools of clones such the diversity of functional screens represented is maximized within a pool. In this way, the presence of homologous genes may be reduced.

One other consideration for the pooled sequencing strategy relates to the issue of consensus assemblies, which may occur for overlapping clones during assembly process (**Figure 6**). Since overlapping clones likely (though not always) result from the same functional screen, it is possible for the experimental biologist to minimize their presence by doing restriction profile comparisons prior to selecting clones for pooling and sequencing. It may also be possible to reduce loss of clone-specific sequence variation by using combinatorial or overlapping clone pooling approaches, which have been used by others for strategic sequencing of BAC clones from genomic libraries [50], [52] as well as plasmid-based oligonucleotide libraries [53]. In such an approach, a large set of clones is divided into subpools such that each clone is present in multiple subpools, but no two clones are in the same subpool more than once, which can help resolve ambiguity in the case that clones in one pool have sequence similarity. In the simplest approach for combining the barcoded and pooled sequencing strategies, a large pool of clones could be split into smaller subpools, each of which gets barcoded. By strategically using a mixture of barcoding, pooling, and/or duplicate sequencing, one can strike a balance between making use of sequencing power and being able to recover accurate and complete clone sequence information.

## Conclusions

In this study, we explored a more economical sequencing strategy than barcoded sequencing, without having to compromise data quality. We used a pooled sequencing method that successfully obtained sequence information for a set of large-insert clones. In particular, we validated this method by comparing the sequence data to reference data generated from barcoded sequencing of the same set of clones. By observing identity and coverage between the two datasets for 73 clones, we demonstrate high quality assemblies from pooled sequence datasets. Using the pooled strategy, retrieved clone sequences showed high accuracy, with identity at 99.9–100% for the majority of clones. The amount of sequence recovered for each clone, however, was variable; averaged across 73 clones, the retrieved coverage was 71%, with some clones showing full coverage, and others with minimal coverage. To estimate actual coverage, we accounted for sequencing gaps, and with this correction, the average coverage increased to 85%. Our results suggest that increasing sequencing depth can improve clone coverage, but that clones that have sequence similarity are problematic in a pooled strategy regardless.

Though pooled sequencing has generated promising results, we acknowledge that refinement of the method is required. In particular, sequencing depth will need to be optimized to obtain maximum recovery of clone sequence, and the choice of clones to pool for sequencing will also need careful consideration, to minimize the presence of clones with sequence similarity. Our results demonstrate that, with further optimization, a pooled sequencing approach could become the preferred method of generating clone sequence data, as its cost is a fraction of that of barcoded sequencing. It is important to note that clone sequence recovery may not be complete or even possible for all clones that have been pooled for sequencing; however, we maintain that until the cost of barcoding many samples becomes affordable in the way that Sanger sequencing has become affordable, pooled sequencing of large sets of clones remains a relevant and reasonable strategy.

## Supporting Information

Figure S1 **Fraction of clones failing assembly, binned by estimated percent** ***E. coli*** **contamination.** Raw sequence data from barcoded sequencing of 92 clones were examined for *E. coli* contamination. Clones were binned by percent contamination, and the fraction of unsuccessfully assembled clones in each bin was calculated.
(EPS)

Figure S2 **Boxplot of clone read depth in barcoded sequencing compared to pooled sequencing**. Values from Table S9 were used to compare overall read depth for barcoded versus pooled sequencing strategies.
(EPS)

Figure S3 **Clone read depth versus clone coverage, in pooled sequencing**. Clone read depth versus uncorrected coverage (A) and corrected coverage (B) are shown.
(EPS)

Table S1 **Functional screens.** List of 92 cosmid clones used in this study, and the corresponding functional screens and metagenomic libraries from which they were isolated (note that NCBI BioSample IDs for the libraries are given in Table 1). Original clone names are shown. Clones that were excluded from analysis due to incomplete sequencing data are indicated.
(XLSX)

Table S2 **Barcodes used in Illumina sequencing.** List of the 92 barcodes used in this study to index clones, to generate the reference sequence data.
(XLSX)

Table S3 **Sanger end-tag sequences.** Forward and/or reverse reads from Sanger-sequencing of the 73 clones analyzed in this study.
(XLSX)

Table S4 **Hit table of retrieved contigs from pooled sequencing results.** Retrieved contigs using forward and/or reverse Sanger end-tags, for each of the 73 clones analyzed in this study. For each clone, results for both end-tags are shown. Columns indicate whether the end-tag was obtained, the end-tag length, the retrieved contig ID, the retrieved contig length, and the alignment identity between end-tag and retrieved contig.
(XLSX)

Table S5 **Accession numbers for raw data uploaded to NCBI Sequence Read Archive.** Experiment names and accession numbers have been provided for barcoded sequencing (92 Experiments) and pooled sequencing (1 Experiment).
(XLSX)

Table S6 **Clone type classification.** 73 clones classified as Clone Type A, B, C, or D. Type descriptions are as follows. Type A: end-tags retrieved same contig from pool. Type B: end-tags retrieved two different contigs from the pool but both contigs belong to same clone. Type C: only one of the two end-tags retrieved a contig from the pool. Type D: single end-tag retrieved one contig from pool.
(XLSX)

Table S7 **Percent coverage of pooled sequencing result relative to barcoded sequencing result.** Retrieved coverage (using end-tags as queries) and estimated actual coverage (using the barcoded sequencing results as queries) are shown for each of the 73 clones analyzed.
(XLSX)

Table S8 **Cost and coverage comparisons for the sequencing strategies used.** A rough breakdown of the cost for both barcoded sequencing and pooled sequencing is shown, rounded to the nearest hundred dollars for each item.
(XLSX)

Table S9 **Clone read depth in barcoded sequencing and pooled sequencing.** The read depth of each of the 73 clones was estimated by comparing raw reads to the barcoded reference sequence.
(XLSX)

File S1 **Estimated sequencing read depth across all clones.** The read depth was plotted across each of the 73 clones for both barcoded and pooled sequencing. Read depth was estimated by comparing raw reads to the barcoded reference sequence.
(ZIP)
