# Supplementary figures and images for: Evaluation of a Pooled Strategy for High-Throughput Sequencing of Cosmid Clones from Metagenomic Libraries
Source: PLoS One. 2014 Jun 9;9(6):e98968. doi: 10.1371/journal.pone.0098968 (PMC4049660; doi:10.1371/journal.pone.0098968)

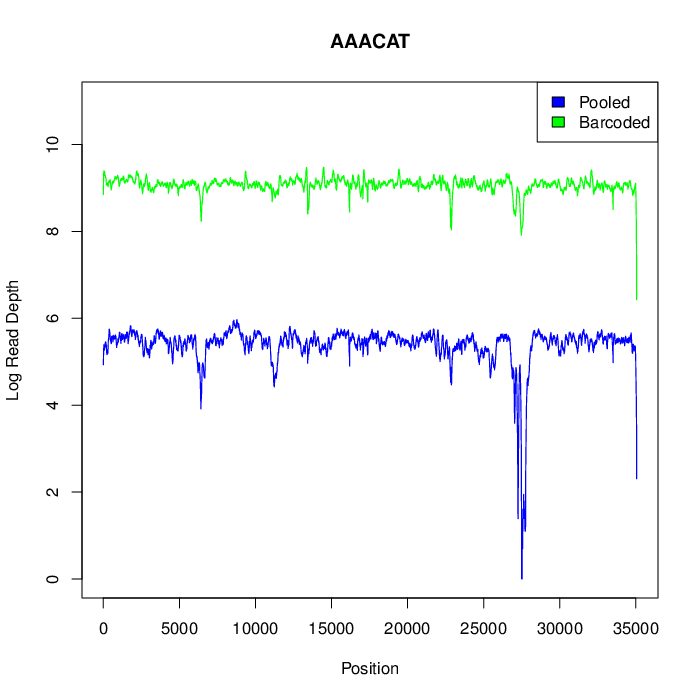

Supplement: File S1 — Estimated sequencing read depth across all clones. The read depth was plotted across each of the 73 clones for both barcoded and pooled sequencing. Read depth was estimated by comparing raw reads to the barcoded reference sequence. (ZIP) [file pone.0098968.s013.zip › AAACAT.png]

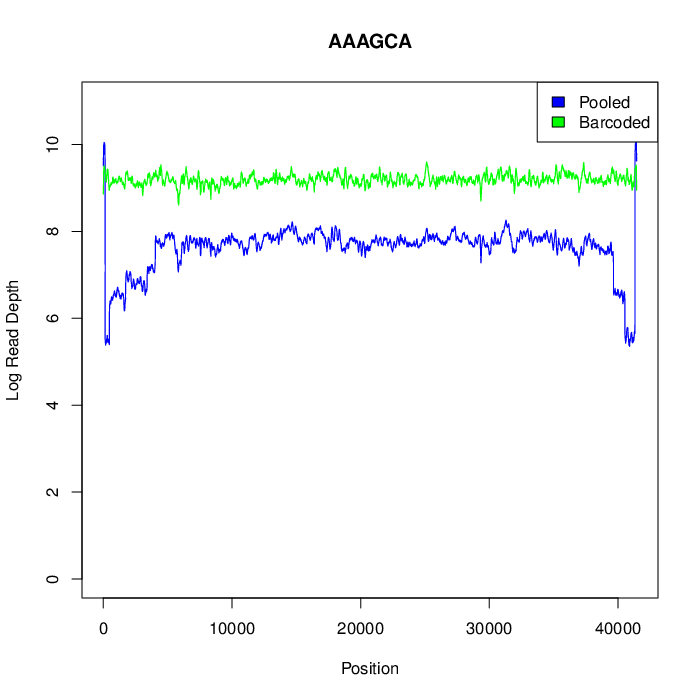

Supplement: File S1 — Estimated sequencing read depth across all clones. The read depth was plotted across each of the 73 clones for both barcoded and pooled sequencing. Read depth was estimated by comparing raw reads to the barcoded reference sequence. (ZIP) [file pone.0098968.s013.zip › AAAGCA.png]

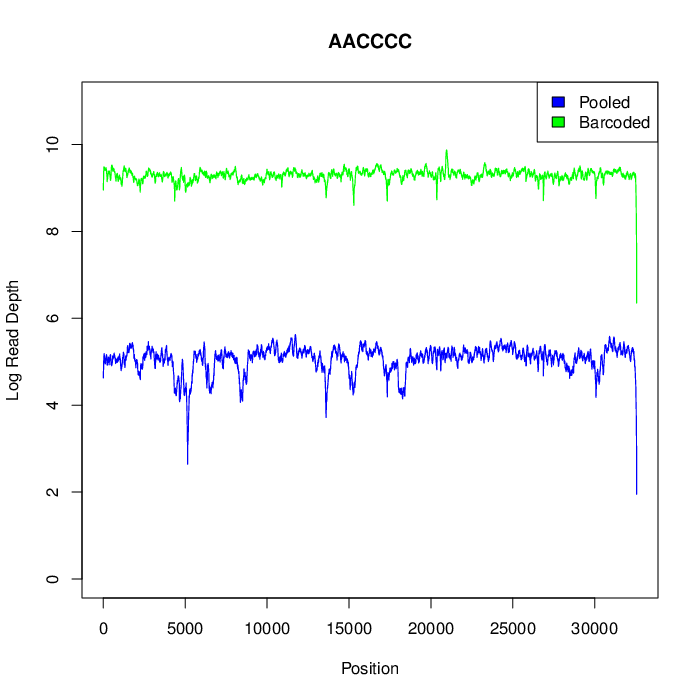

Supplement: File S1 — Estimated sequencing read depth across all clones. The read depth was plotted across each of the 73 clones for both barcoded and pooled sequencing. Read depth was estimated by comparing raw reads to the barcoded reference sequence. (ZIP) [file pone.0098968.s013.zip › AACCCC.png]

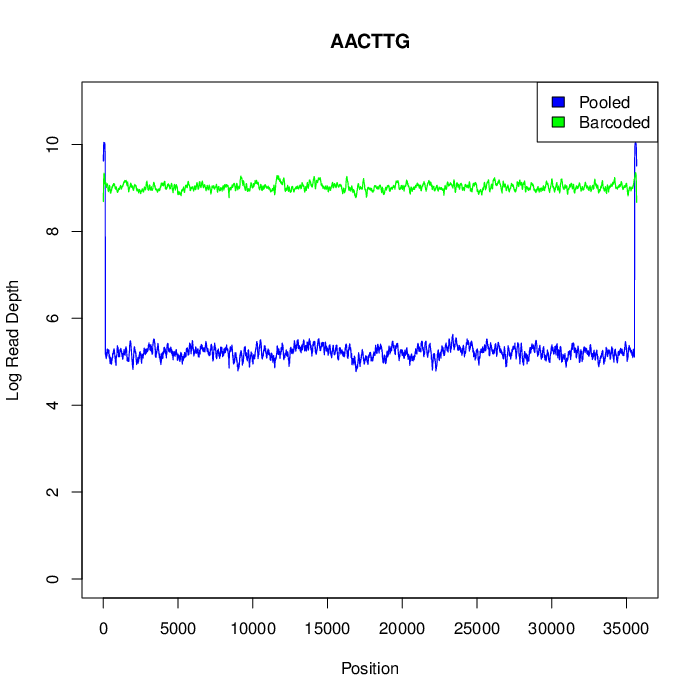

Supplement: File S1 — Estimated sequencing read depth across all clones. The read depth was plotted across each of the 73 clones for both barcoded and pooled sequencing. Read depth was estimated by comparing raw reads to the barcoded reference sequence. (ZIP) [file pone.0098968.s013.zip › AACTTG.png]

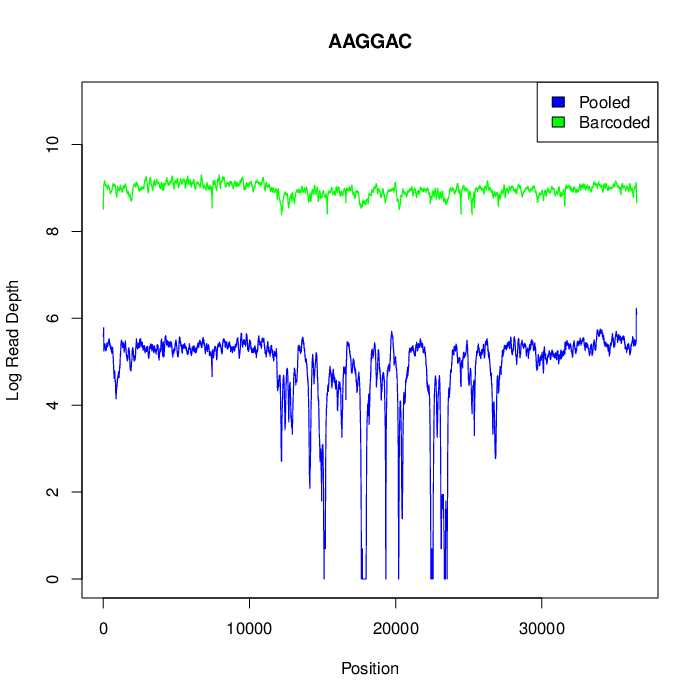

Supplement: File S1 — Estimated sequencing read depth across all clones. The read depth was plotted across each of the 73 clones for both barcoded and pooled sequencing. Read depth was estimated by comparing raw reads to the barcoded reference sequence. (ZIP) [file pone.0098968.s013.zip › AAGGAC.png]

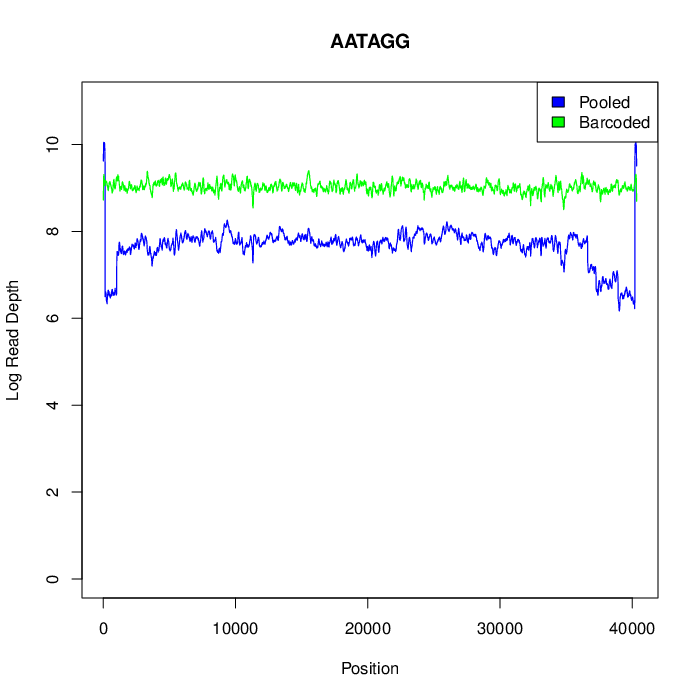

Supplement: File S1 — Estimated sequencing read depth across all clones. The read depth was plotted across each of the 73 clones for both barcoded and pooled sequencing. Read depth was estimated by comparing raw reads to the barcoded reference sequence. (ZIP) [file pone.0098968.s013.zip › AATAGG.png]

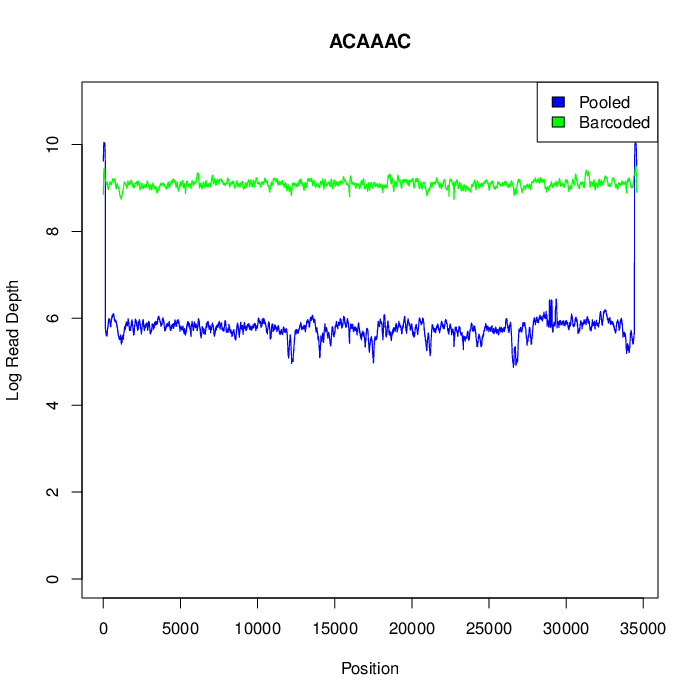

Supplement: File S1 — Estimated sequencing read depth across all clones. The read depth was plotted across each of the 73 clones for both barcoded and pooled sequencing. Read depth was estimated by comparing raw reads to the barcoded reference sequence. (ZIP) [file pone.0098968.s013.zip › ACAAAC.png]

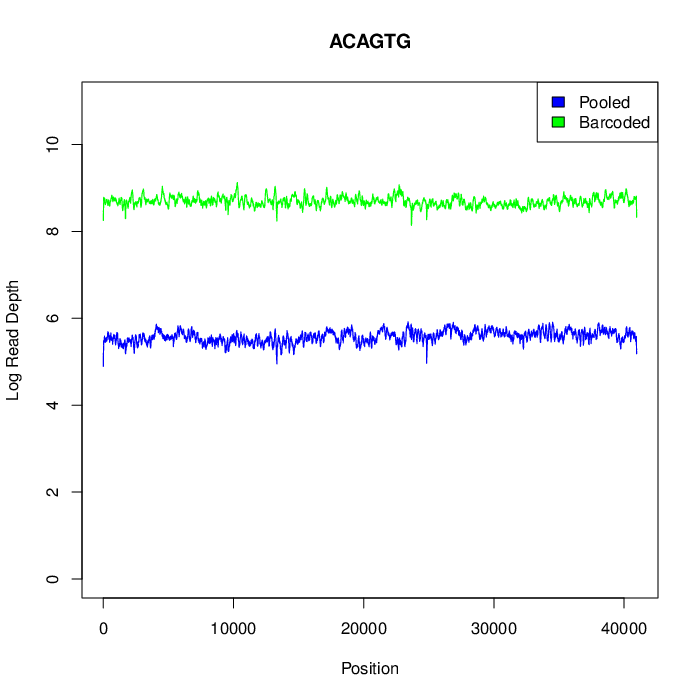

Supplement: File S1 — Estimated sequencing read depth across all clones. The read depth was plotted across each of the 73 clones for both barcoded and pooled sequencing. Read depth was estimated by comparing raw reads to the barcoded reference sequence. (ZIP) [file pone.0098968.s013.zip › ACAGTG.png]

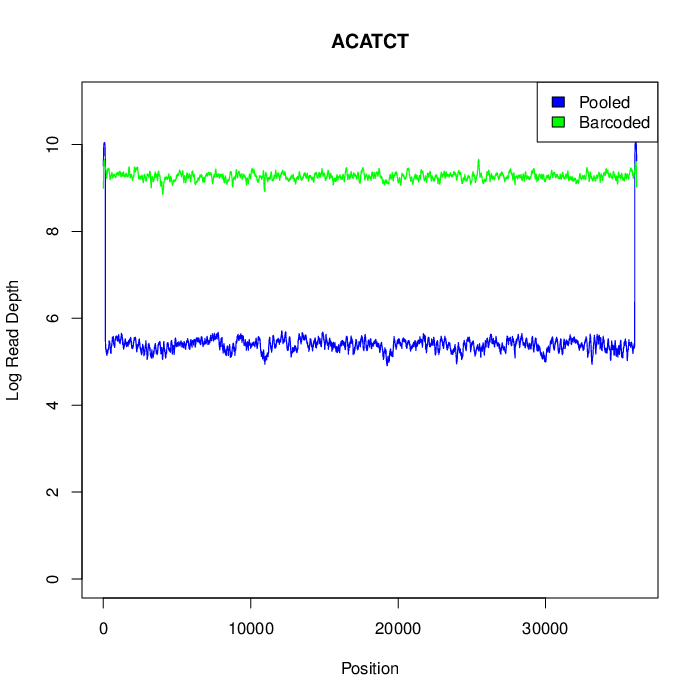

Supplement: File S1 — Estimated sequencing read depth across all clones. The read depth was plotted across each of the 73 clones for both barcoded and pooled sequencing. Read depth was estimated by comparing raw reads to the barcoded reference sequence. (ZIP) [file pone.0098968.s013.zip › ACATCT.png]

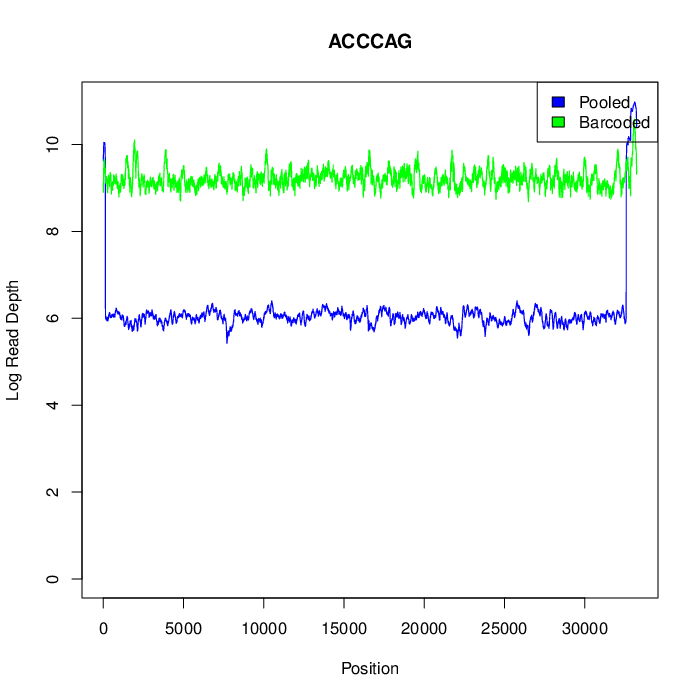

Supplement: File S1 — Estimated sequencing read depth across all clones. The read depth was plotted across each of the 73 clones for both barcoded and pooled sequencing. Read depth was estimated by comparing raw reads to the barcoded reference sequence. (ZIP) [file pone.0098968.s013.zip › ACCCAG.png]

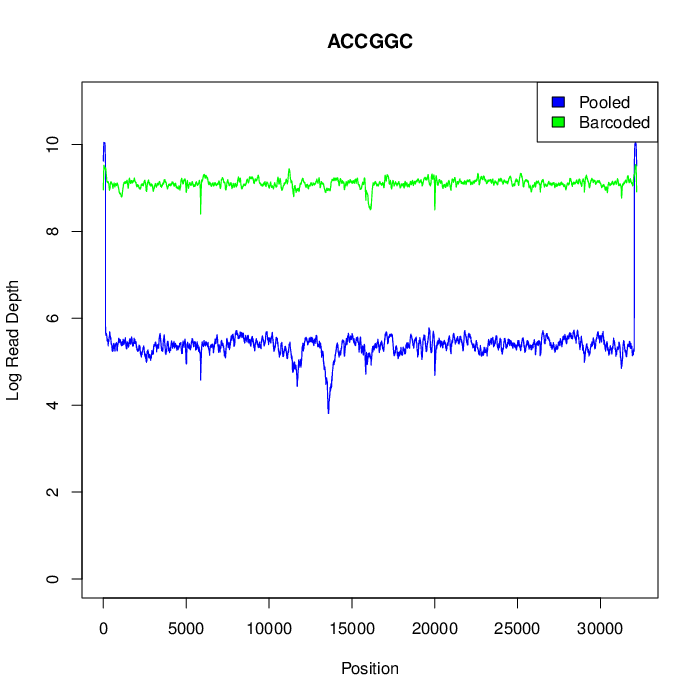

Supplement: File S1 — Estimated sequencing read depth across all clones. The read depth was plotted across each of the 73 clones for both barcoded and pooled sequencing. Read depth was estimated by comparing raw reads to the barcoded reference sequence. (ZIP) [file pone.0098968.s013.zip › ACCGGC.png]

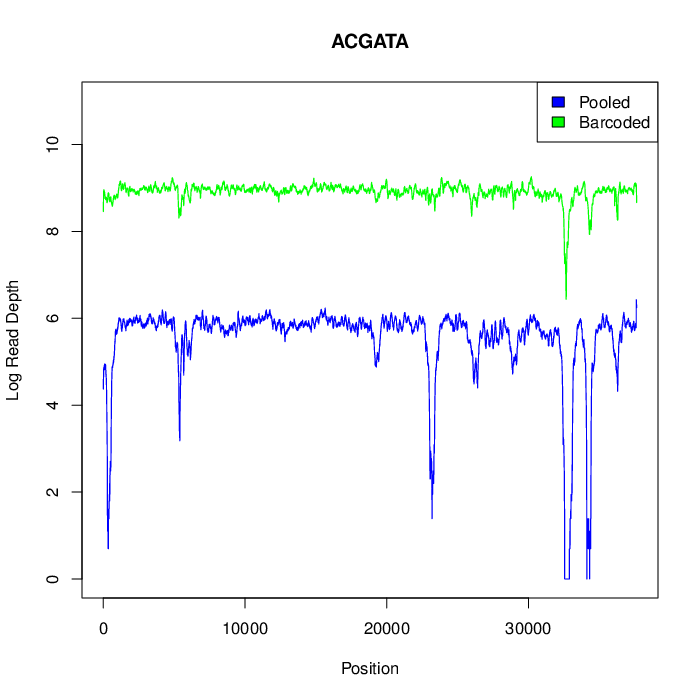

Supplement: File S1 — Estimated sequencing read depth across all clones. The read depth was plotted across each of the 73 clones for both barcoded and pooled sequencing. Read depth was estimated by comparing raw reads to the barcoded reference sequence. (ZIP) [file pone.0098968.s013.zip › ACGATA.png]

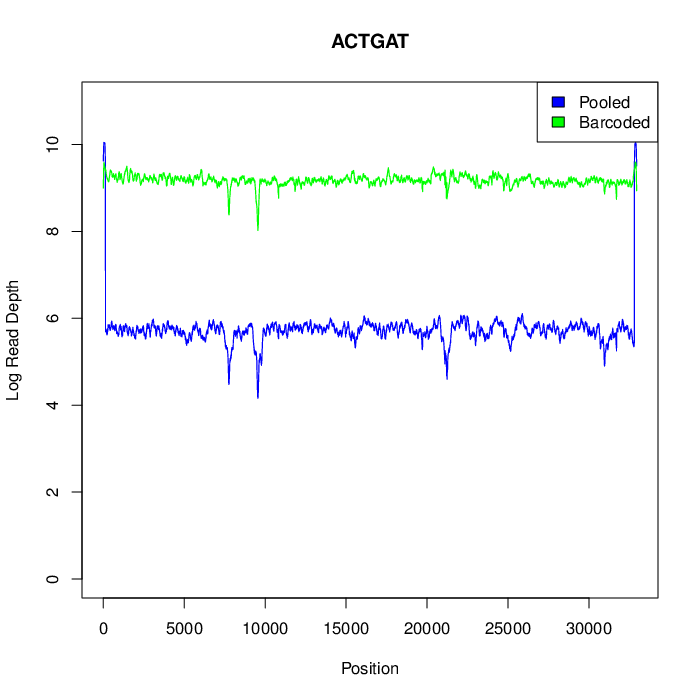

Supplement: File S1 — Estimated sequencing read depth across all clones. The read depth was plotted across each of the 73 clones for both barcoded and pooled sequencing. Read depth was estimated by comparing raw reads to the barcoded reference sequence. (ZIP) [file pone.0098968.s013.zip › ACTGAT.png]

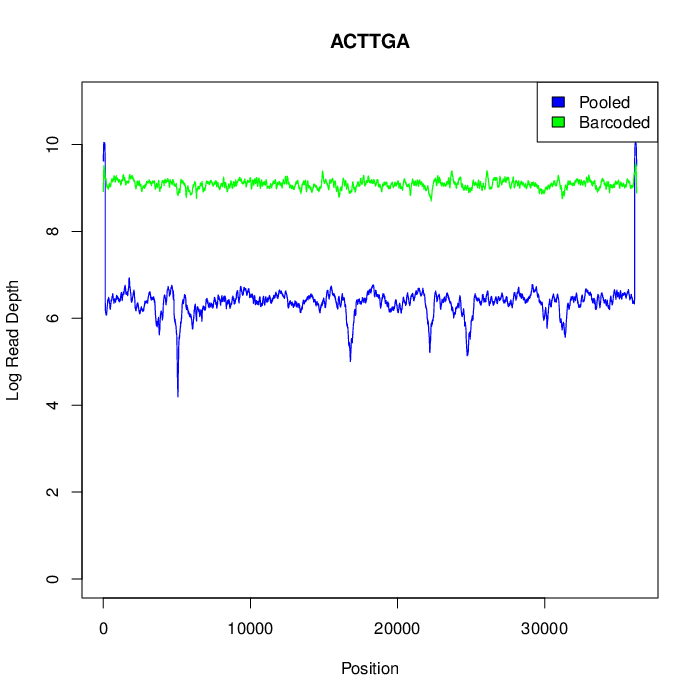

Supplement: File S1 — Estimated sequencing read depth across all clones. The read depth was plotted across each of the 73 clones for both barcoded and pooled sequencing. Read depth was estimated by comparing raw reads to the barcoded reference sequence. (ZIP) [file pone.0098968.s013.zip › ACTTGA.png]

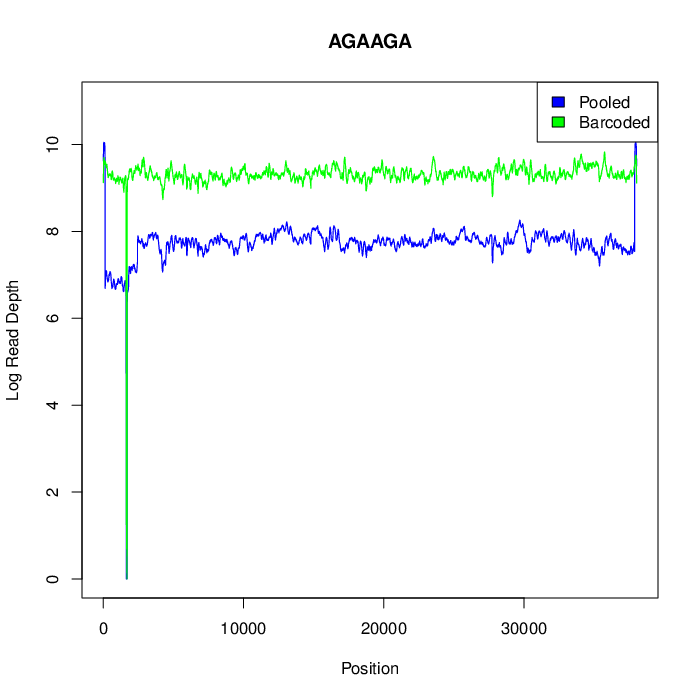

Supplement: File S1 — Estimated sequencing read depth across all clones. The read depth was plotted across each of the 73 clones for both barcoded and pooled sequencing. Read depth was estimated by comparing raw reads to the barcoded reference sequence. (ZIP) [file pone.0098968.s013.zip › AGAAGA.png]

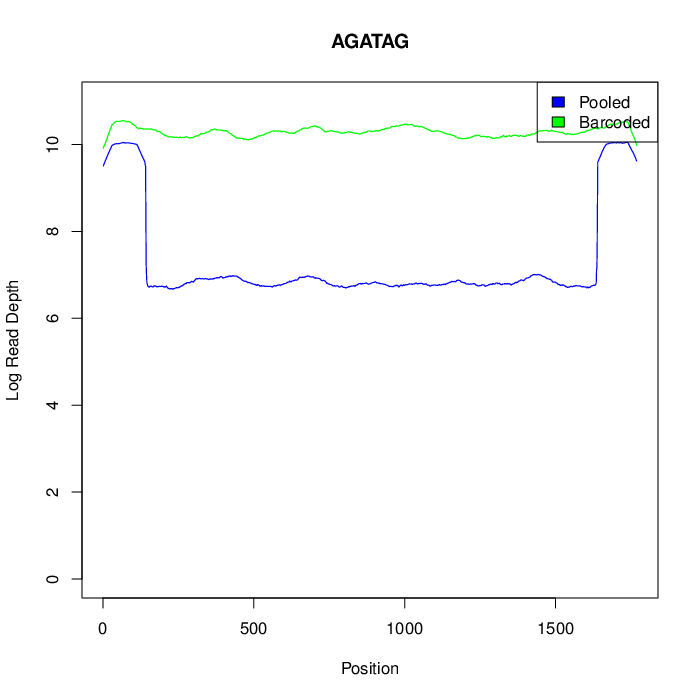

Supplement: File S1 — Estimated sequencing read depth across all clones. The read depth was plotted across each of the 73 clones for both barcoded and pooled sequencing. Read depth was estimated by comparing raw reads to the barcoded reference sequence. (ZIP) [file pone.0098968.s013.zip › AGATAG.png]

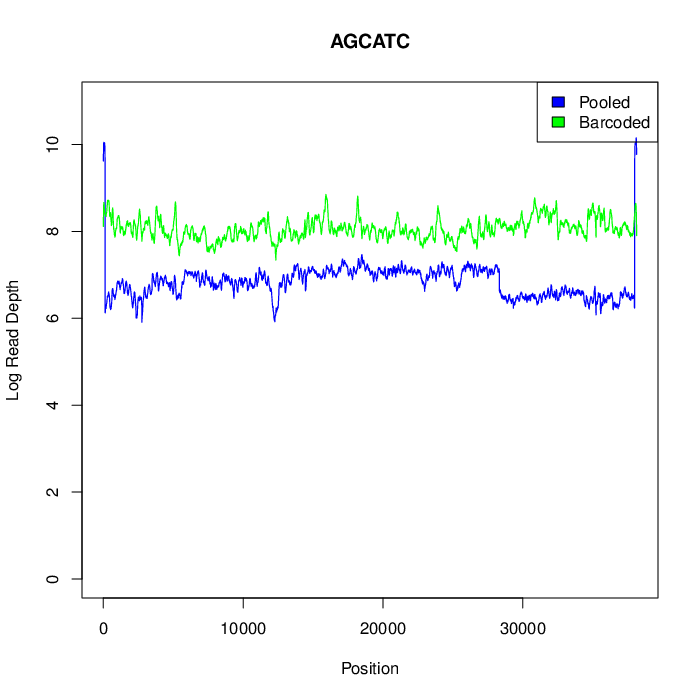

Supplement: File S1 — Estimated sequencing read depth across all clones. The read depth was plotted across each of the 73 clones for both barcoded and pooled sequencing. Read depth was estimated by comparing raw reads to the barcoded reference sequence. (ZIP) [file pone.0098968.s013.zip › AGCATC.png]

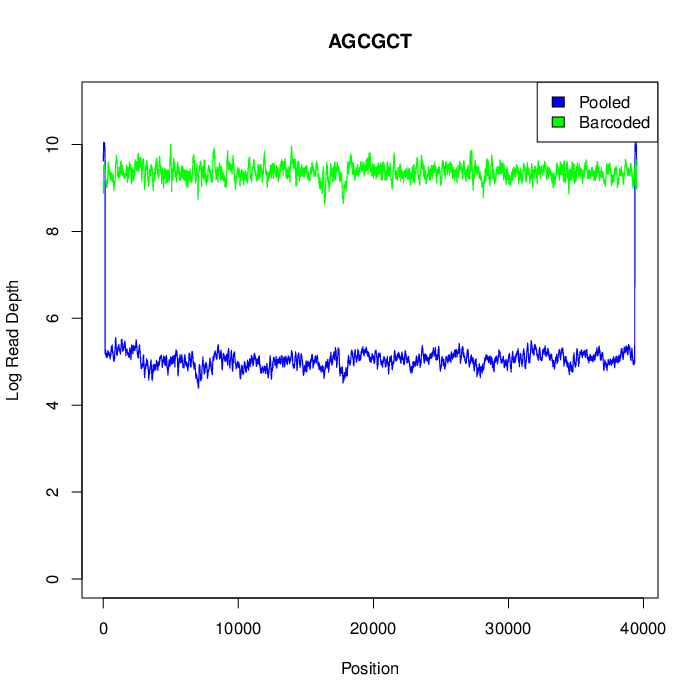

Supplement: File S1 — Estimated sequencing read depth across all clones. The read depth was plotted across each of the 73 clones for both barcoded and pooled sequencing. Read depth was estimated by comparing raw reads to the barcoded reference sequence. (ZIP) [file pone.0098968.s013.zip › AGCGCT.png]

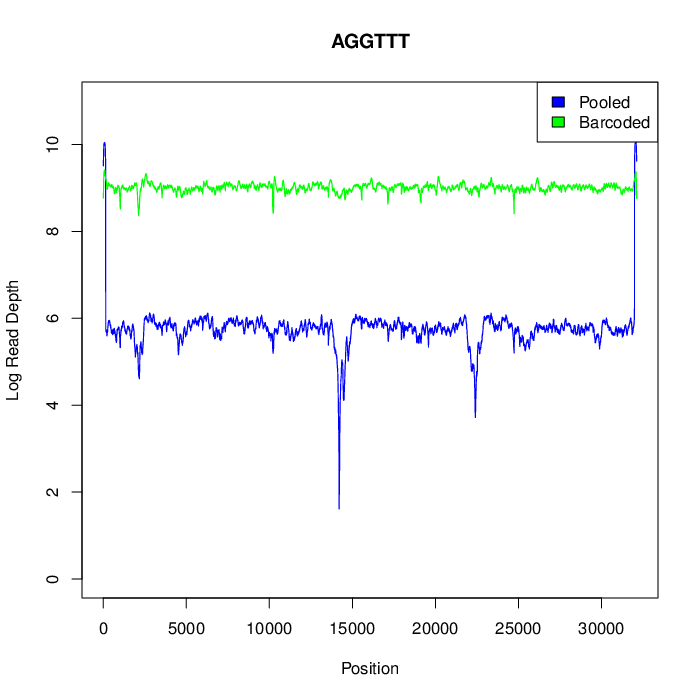

Supplement: File S1 — Estimated sequencing read depth across all clones. The read depth was plotted across each of the 73 clones for both barcoded and pooled sequencing. Read depth was estimated by comparing raw reads to the barcoded reference sequence. (ZIP) [file pone.0098968.s013.zip › AGGTTT.png]

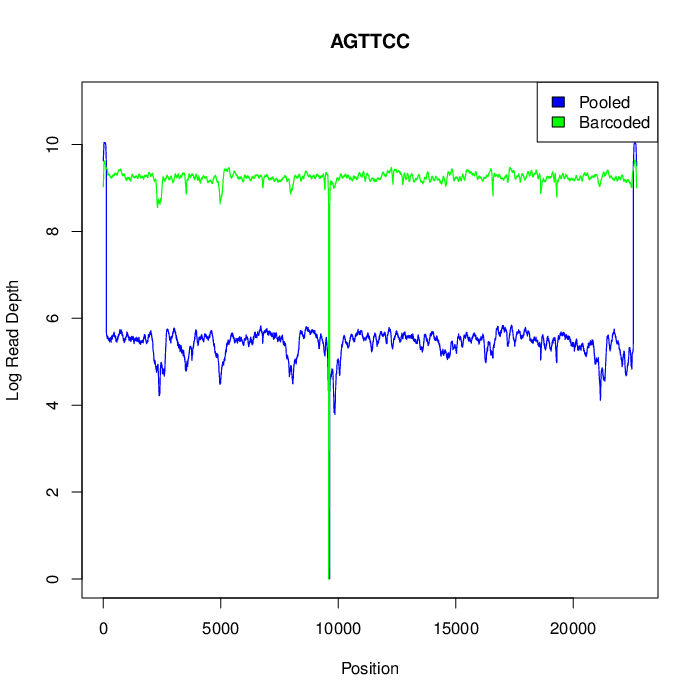

Supplement: File S1 — Estimated sequencing read depth across all clones. The read depth was plotted across each of the 73 clones for both barcoded and pooled sequencing. Read depth was estimated by comparing raw reads to the barcoded reference sequence. (ZIP) [file pone.0098968.s013.zip › AGTTCC.png]

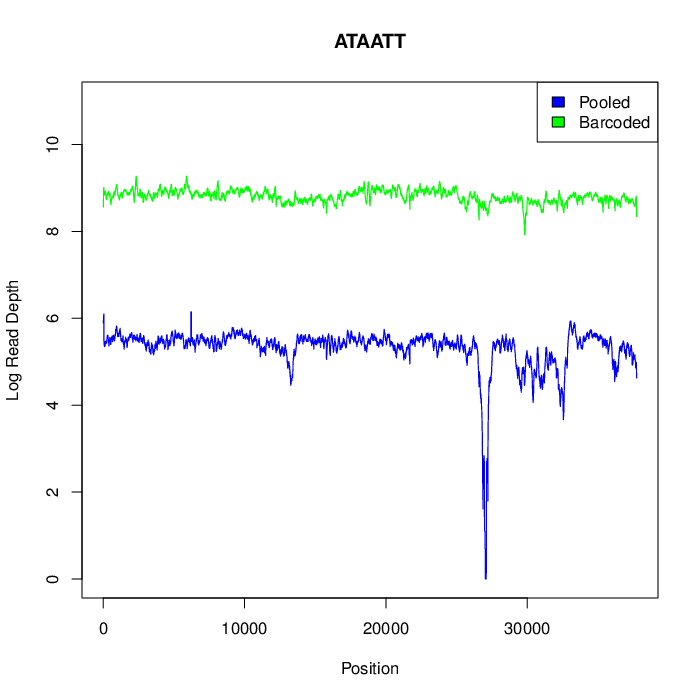

Supplement: File S1 — Estimated sequencing read depth across all clones. The read depth was plotted across each of the 73 clones for both barcoded and pooled sequencing. Read depth was estimated by comparing raw reads to the barcoded reference sequence. (ZIP) [file pone.0098968.s013.zip › ATAATT.png]

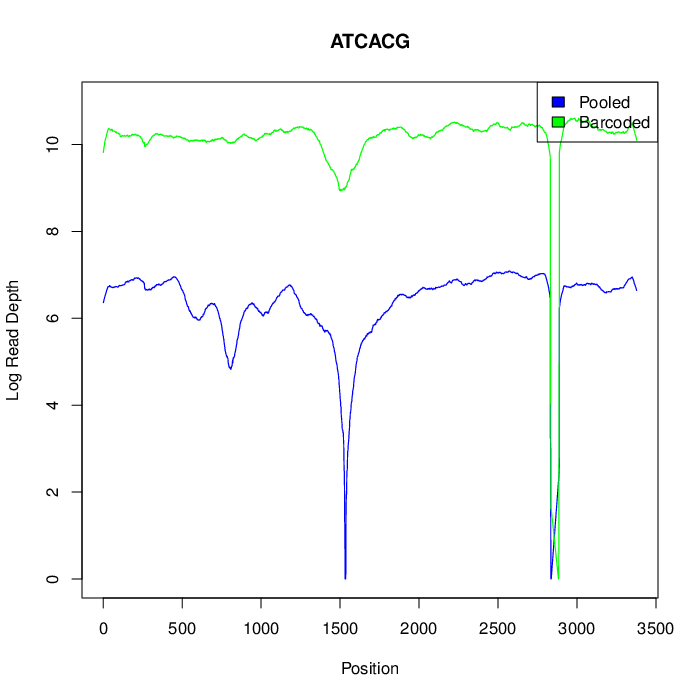

Supplement: File S1 — Estimated sequencing read depth across all clones. The read depth was plotted across each of the 73 clones for both barcoded and pooled sequencing. Read depth was estimated by comparing raw reads to the barcoded reference sequence. (ZIP) [file pone.0098968.s013.zip › ATCACG.png]

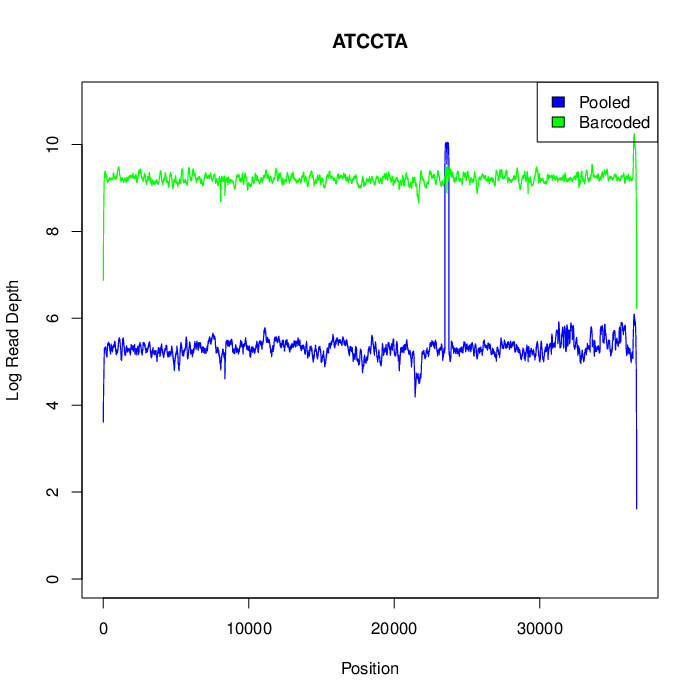

Supplement: File S1 — Estimated sequencing read depth across all clones. The read depth was plotted across each of the 73 clones for both barcoded and pooled sequencing. Read depth was estimated by comparing raw reads to the barcoded reference sequence. (ZIP) [file pone.0098968.s013.zip › ATCCTA.png]

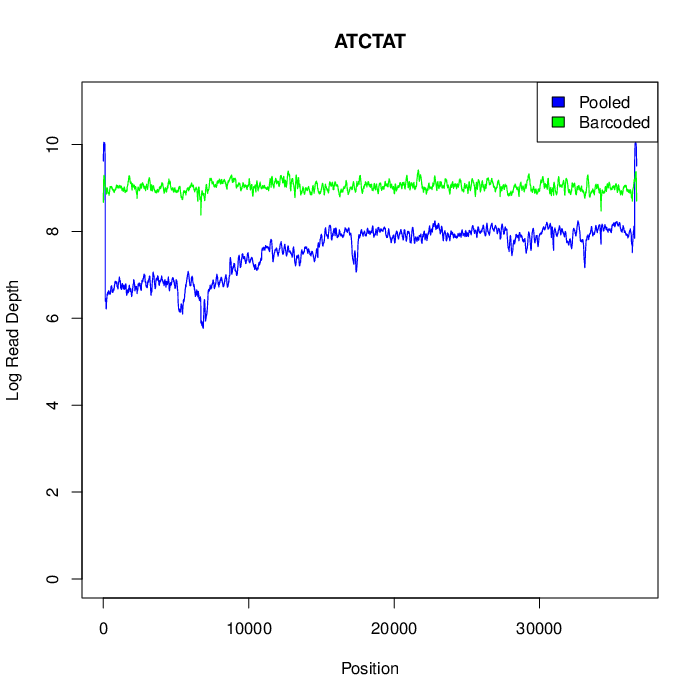

Supplement: File S1 — Estimated sequencing read depth across all clones. The read depth was plotted across each of the 73 clones for both barcoded and pooled sequencing. Read depth was estimated by comparing raw reads to the barcoded reference sequence. (ZIP) [file pone.0098968.s013.zip › ATCTAT.png]

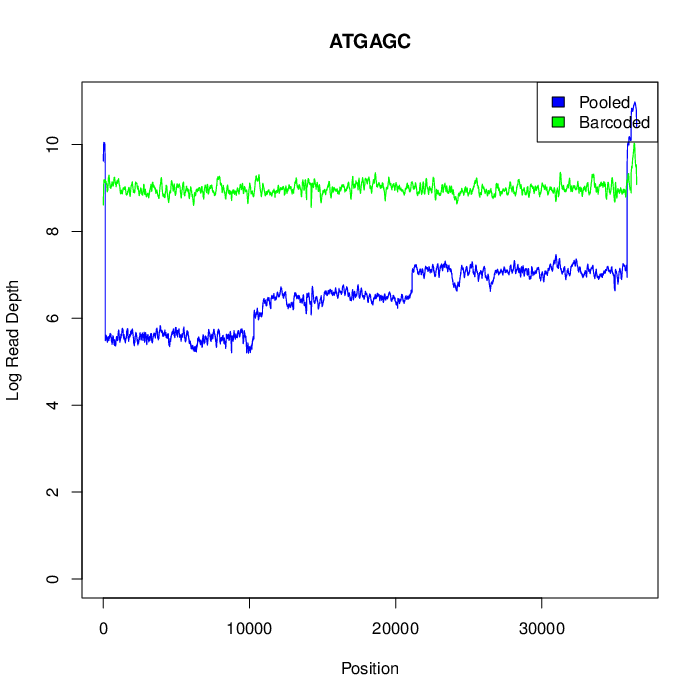

Supplement: File S1 — Estimated sequencing read depth across all clones. The read depth was plotted across each of the 73 clones for both barcoded and pooled sequencing. Read depth was estimated by comparing raw reads to the barcoded reference sequence. (ZIP) [file pone.0098968.s013.zip › ATGAGC.png]

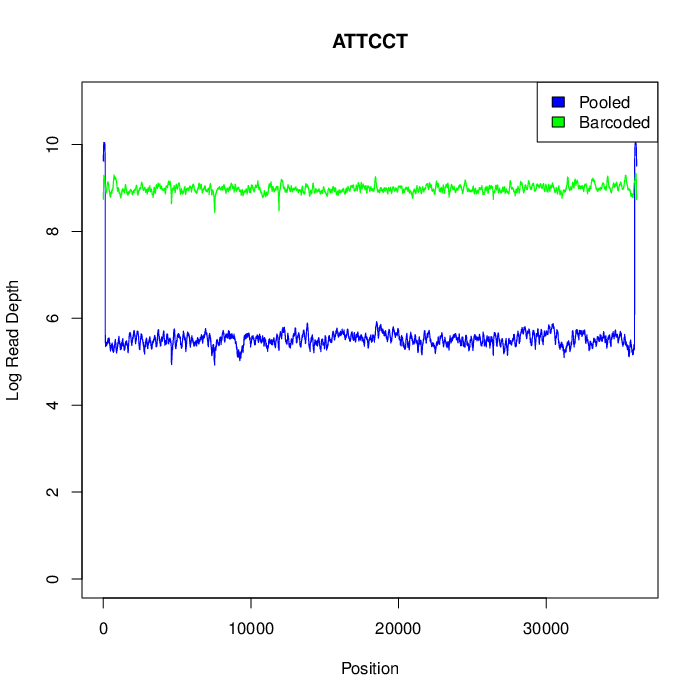

Supplement: File S1 — Estimated sequencing read depth across all clones. The read depth was plotted across each of the 73 clones for both barcoded and pooled sequencing. Read depth was estimated by comparing raw reads to the barcoded reference sequence. (ZIP) [file pone.0098968.s013.zip › ATTCCT.png]

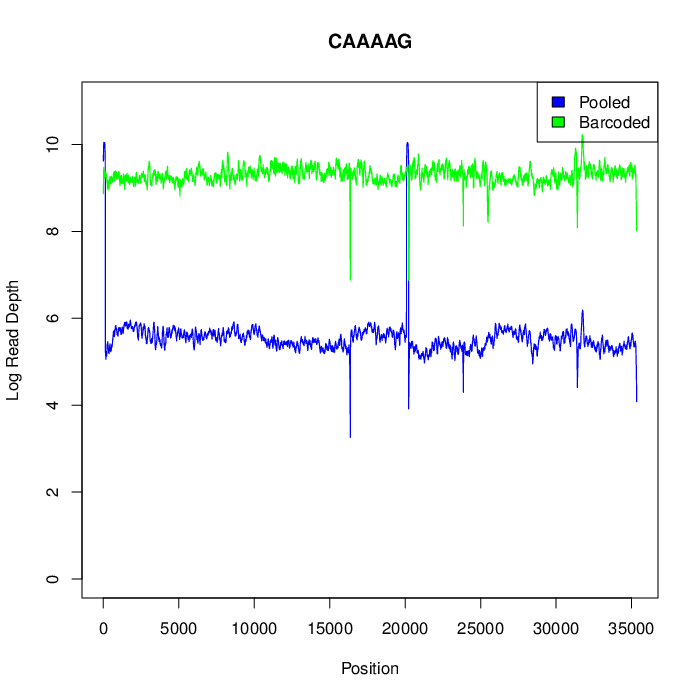

Supplement: File S1 — Estimated sequencing read depth across all clones. The read depth was plotted across each of the 73 clones for both barcoded and pooled sequencing. Read depth was estimated by comparing raw reads to the barcoded reference sequence. (ZIP) [file pone.0098968.s013.zip › CAAAAG.png]

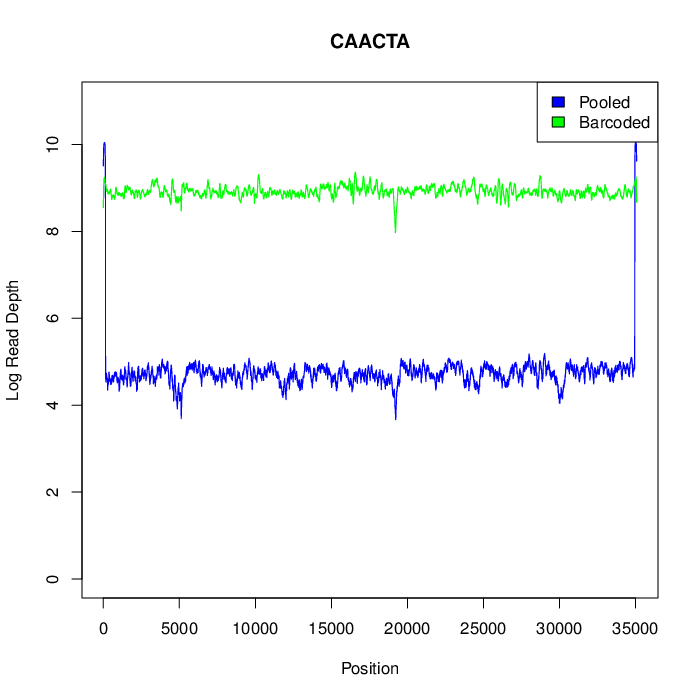

Supplement: File S1 — Estimated sequencing read depth across all clones. The read depth was plotted across each of the 73 clones for both barcoded and pooled sequencing. Read depth was estimated by comparing raw reads to the barcoded reference sequence. (ZIP) [file pone.0098968.s013.zip › CAACTA.png]

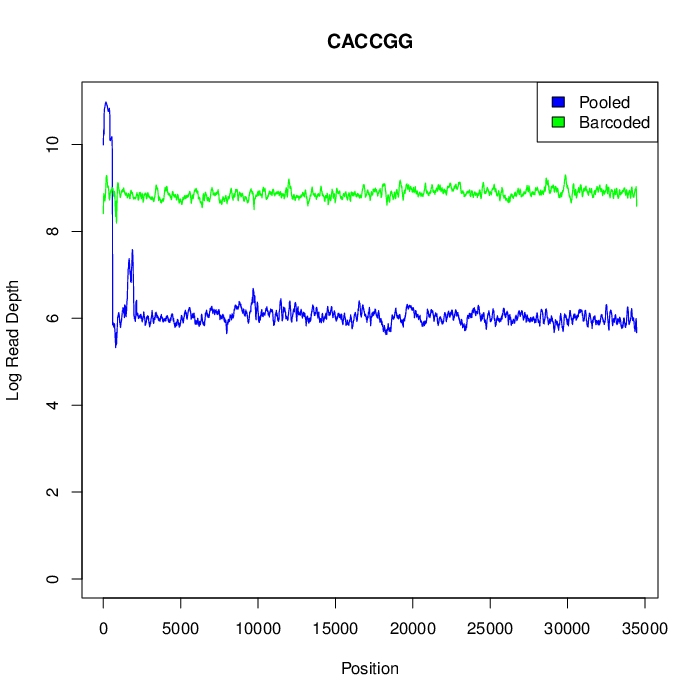

Supplement: File S1 — Estimated sequencing read depth across all clones. The read depth was plotted across each of the 73 clones for both barcoded and pooled sequencing. Read depth was estimated by comparing raw reads to the barcoded reference sequence. (ZIP) [file pone.0098968.s013.zip › CACCGG.png]

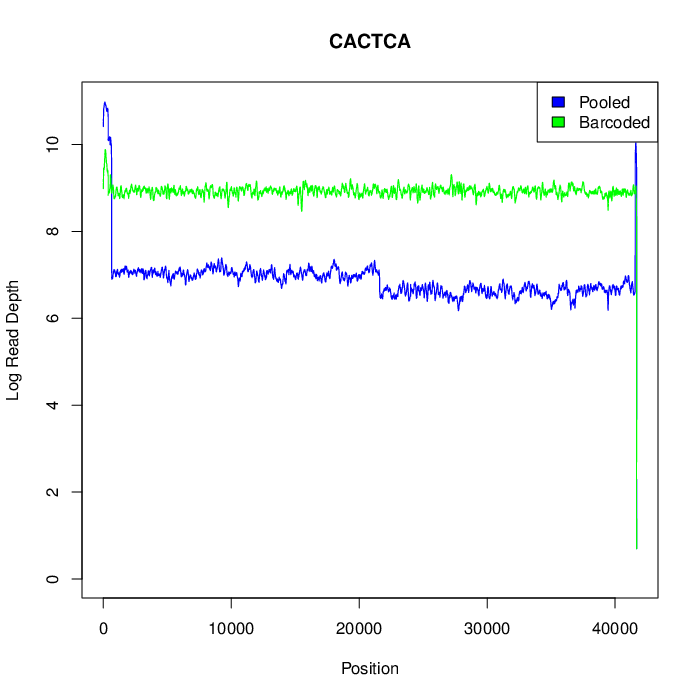

Supplement: File S1 — Estimated sequencing read depth across all clones. The read depth was plotted across each of the 73 clones for both barcoded and pooled sequencing. Read depth was estimated by comparing raw reads to the barcoded reference sequence. (ZIP) [file pone.0098968.s013.zip › CACTCA.png]

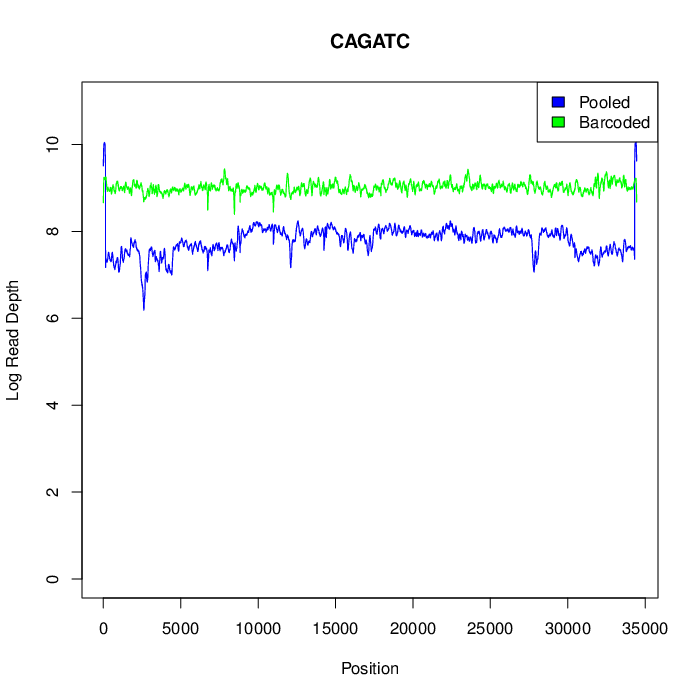

Supplement: File S1 — Estimated sequencing read depth across all clones. The read depth was plotted across each of the 73 clones for both barcoded and pooled sequencing. Read depth was estimated by comparing raw reads to the barcoded reference sequence. (ZIP) [file pone.0098968.s013.zip › CAGATC.png]

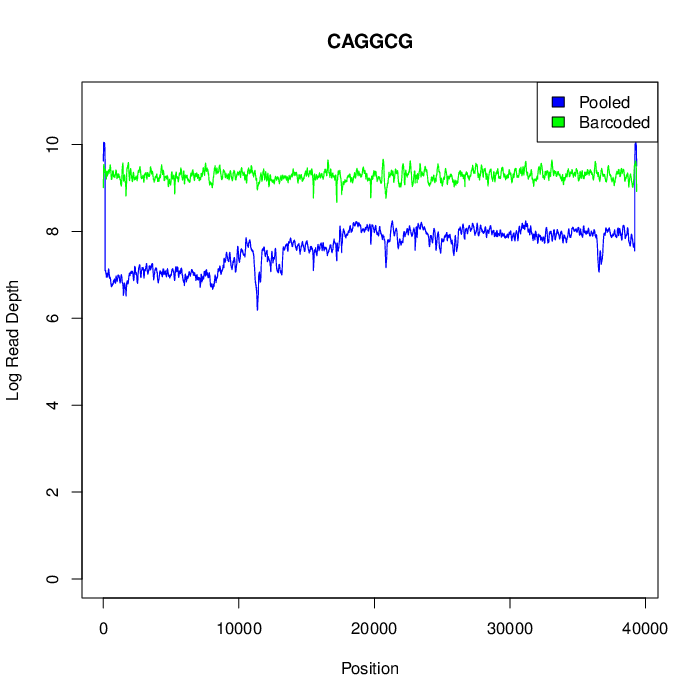

Supplement: File S1 — Estimated sequencing read depth across all clones. The read depth was plotted across each of the 73 clones for both barcoded and pooled sequencing. Read depth was estimated by comparing raw reads to the barcoded reference sequence. (ZIP) [file pone.0098968.s013.zip › CAGGCG.png]

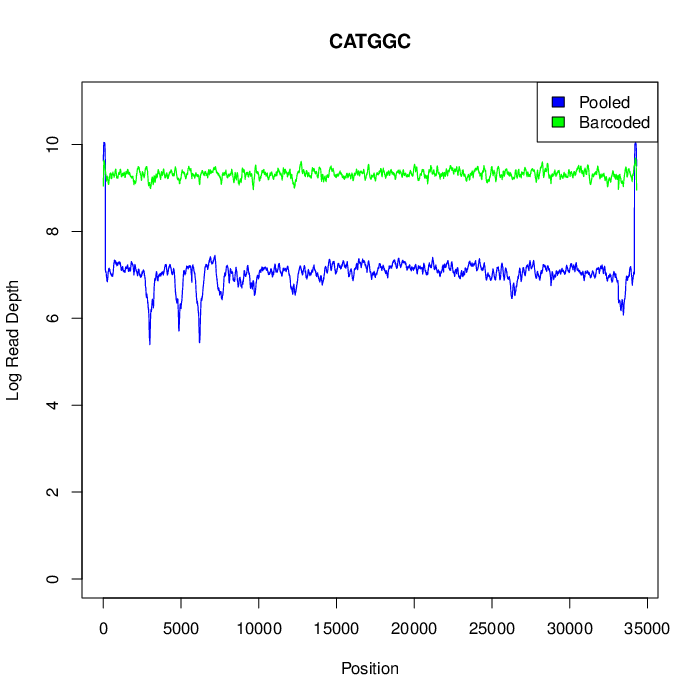

Supplement: File S1 — Estimated sequencing read depth across all clones. The read depth was plotted across each of the 73 clones for both barcoded and pooled sequencing. Read depth was estimated by comparing raw reads to the barcoded reference sequence. (ZIP) [file pone.0098968.s013.zip › CATGGC.png]

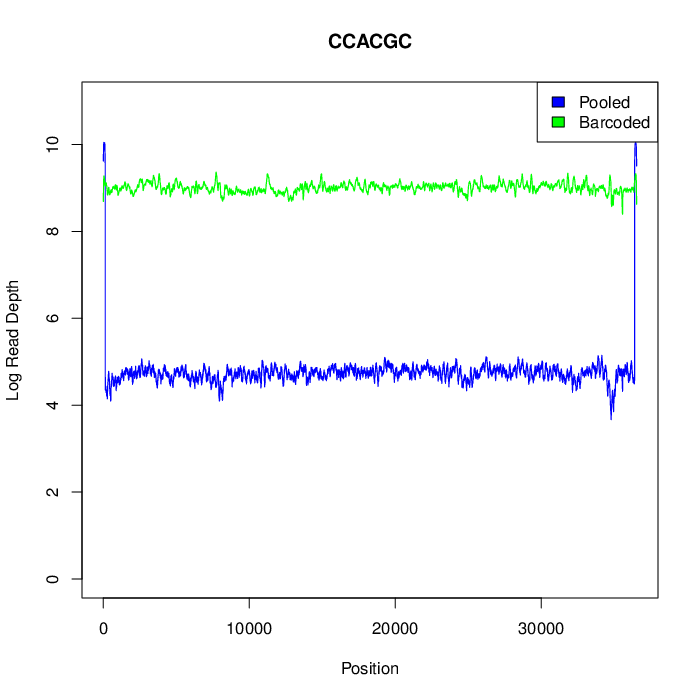

Supplement: File S1 — Estimated sequencing read depth across all clones. The read depth was plotted across each of the 73 clones for both barcoded and pooled sequencing. Read depth was estimated by comparing raw reads to the barcoded reference sequence. (ZIP) [file pone.0098968.s013.zip › CCACGC.png]

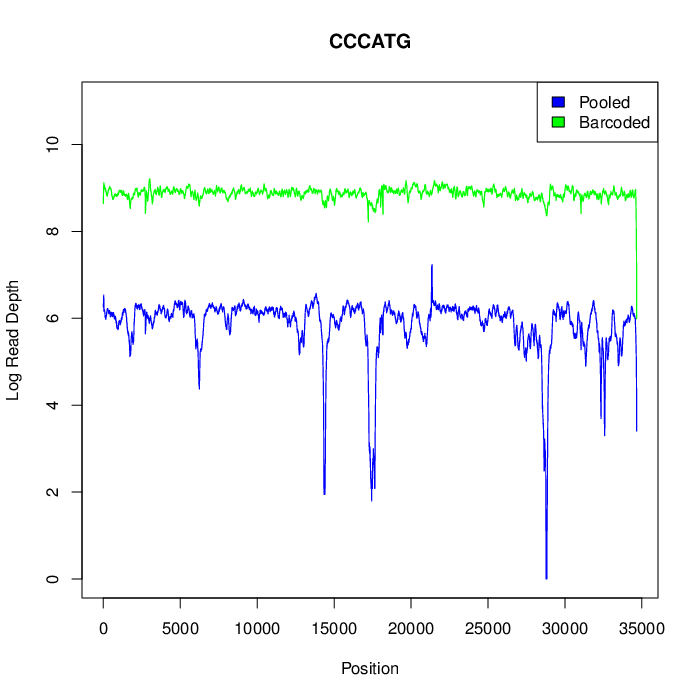

Supplement: File S1 — Estimated sequencing read depth across all clones. The read depth was plotted across each of the 73 clones for both barcoded and pooled sequencing. Read depth was estimated by comparing raw reads to the barcoded reference sequence. (ZIP) [file pone.0098968.s013.zip › CCCATG.png]

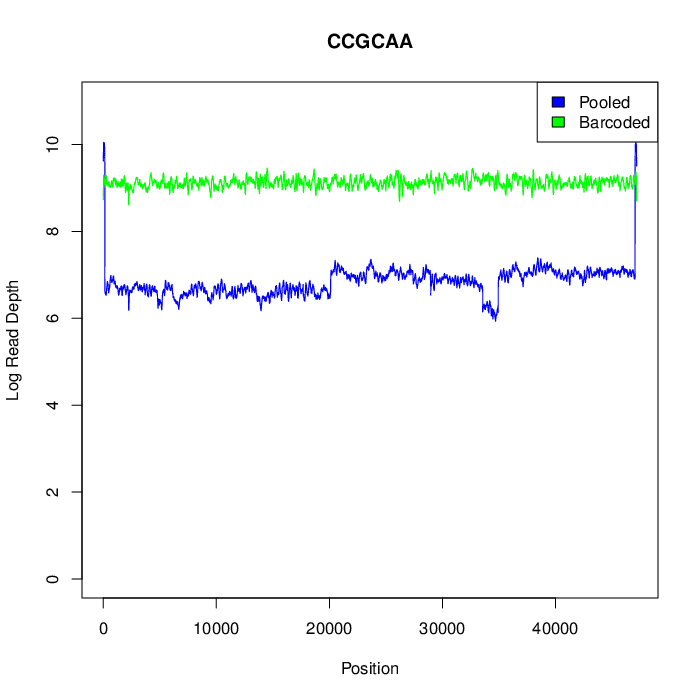

Supplement: File S1 — Estimated sequencing read depth across all clones. The read depth was plotted across each of the 73 clones for both barcoded and pooled sequencing. Read depth was estimated by comparing raw reads to the barcoded reference sequence. (ZIP) [file pone.0098968.s013.zip › CCGCAA.png]

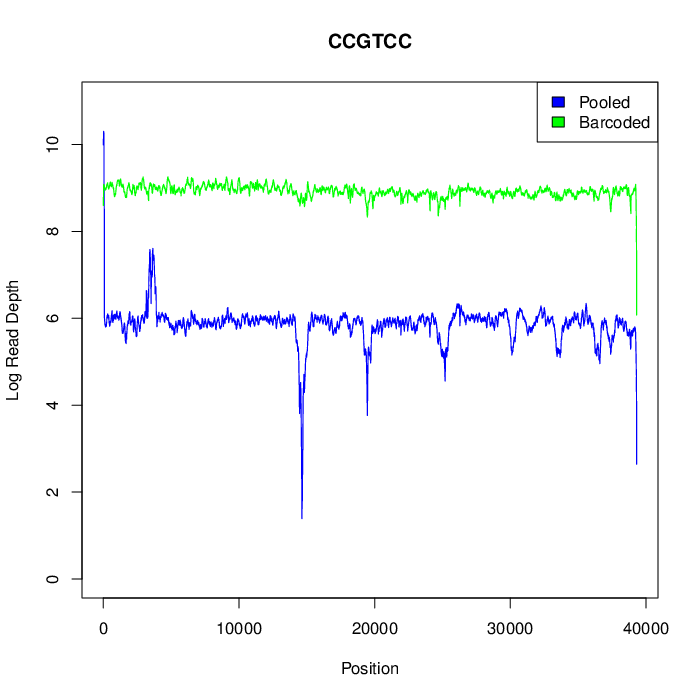

Supplement: File S1 — Estimated sequencing read depth across all clones. The read depth was plotted across each of the 73 clones for both barcoded and pooled sequencing. Read depth was estimated by comparing raw reads to the barcoded reference sequence. (ZIP) [file pone.0098968.s013.zip › CCGTCC.png]

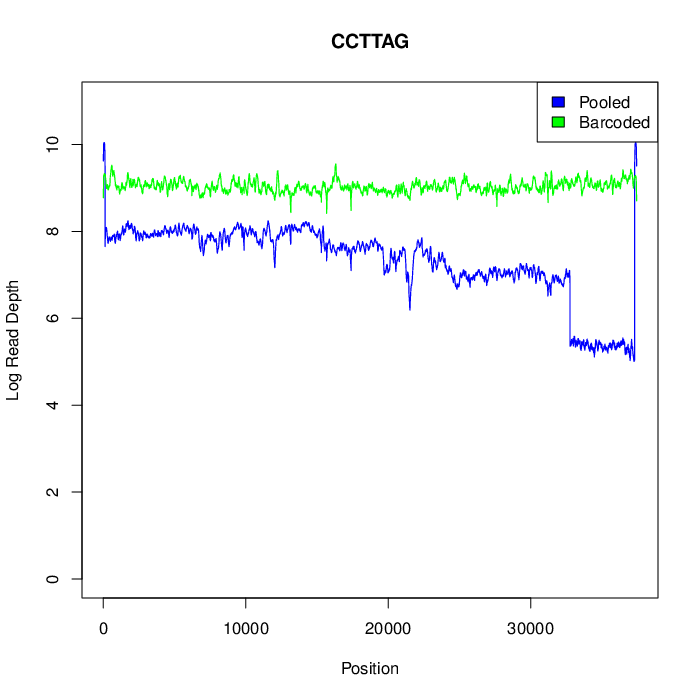

Supplement: File S1 — Estimated sequencing read depth across all clones. The read depth was plotted across each of the 73 clones for both barcoded and pooled sequencing. Read depth was estimated by comparing raw reads to the barcoded reference sequence. (ZIP) [file pone.0098968.s013.zip › CCTTAG.png]

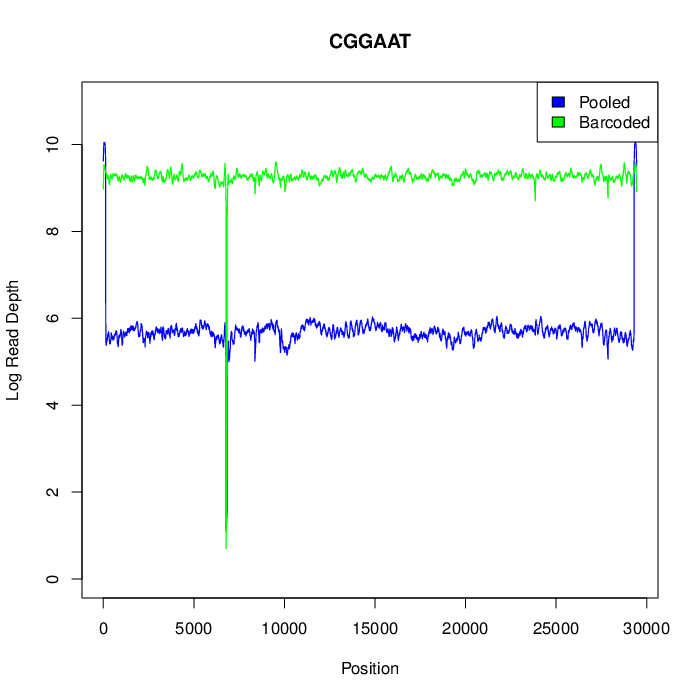

Supplement: File S1 — Estimated sequencing read depth across all clones. The read depth was plotted across each of the 73 clones for both barcoded and pooled sequencing. Read depth was estimated by comparing raw reads to the barcoded reference sequence. (ZIP) [file pone.0098968.s013.zip › CGGAAT.png]

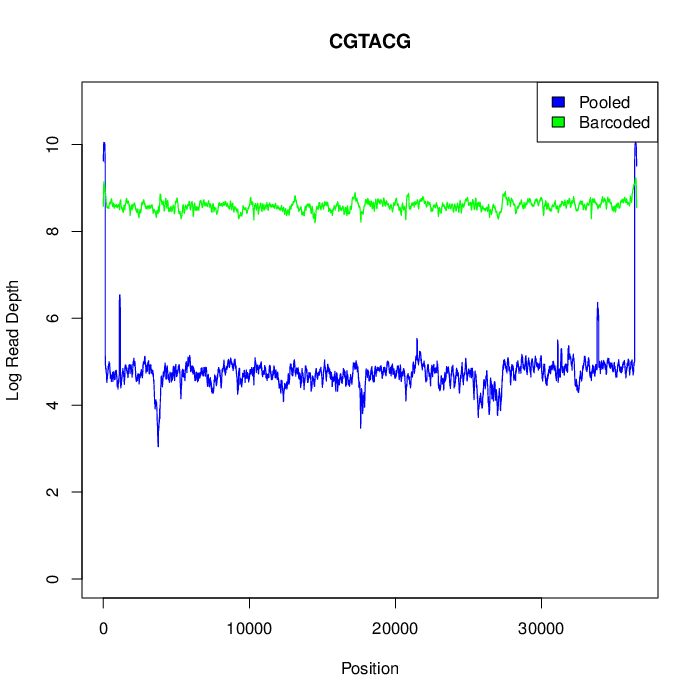

Supplement: File S1 — Estimated sequencing read depth across all clones. The read depth was plotted across each of the 73 clones for both barcoded and pooled sequencing. Read depth was estimated by comparing raw reads to the barcoded reference sequence. (ZIP) [file pone.0098968.s013.zip › CGTACG.png]

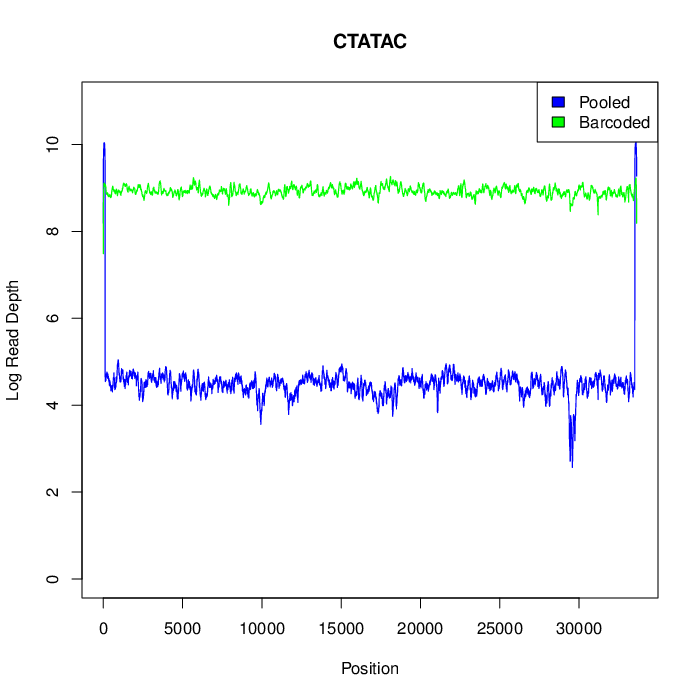

Supplement: File S1 — Estimated sequencing read depth across all clones. The read depth was plotted across each of the 73 clones for both barcoded and pooled sequencing. Read depth was estimated by comparing raw reads to the barcoded reference sequence. (ZIP) [file pone.0098968.s013.zip › CTATAC.png]

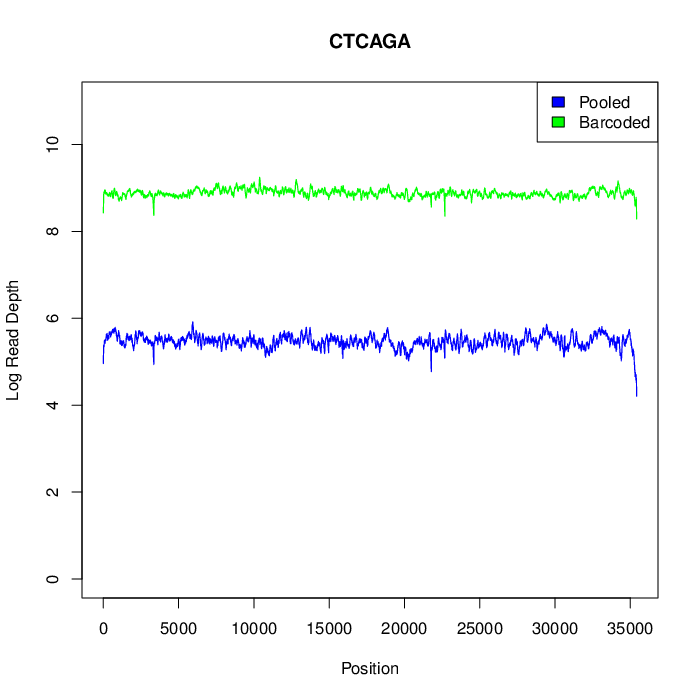

Supplement: File S1 — Estimated sequencing read depth across all clones. The read depth was plotted across each of the 73 clones for both barcoded and pooled sequencing. Read depth was estimated by comparing raw reads to the barcoded reference sequence. (ZIP) [file pone.0098968.s013.zip › CTCAGA.png]

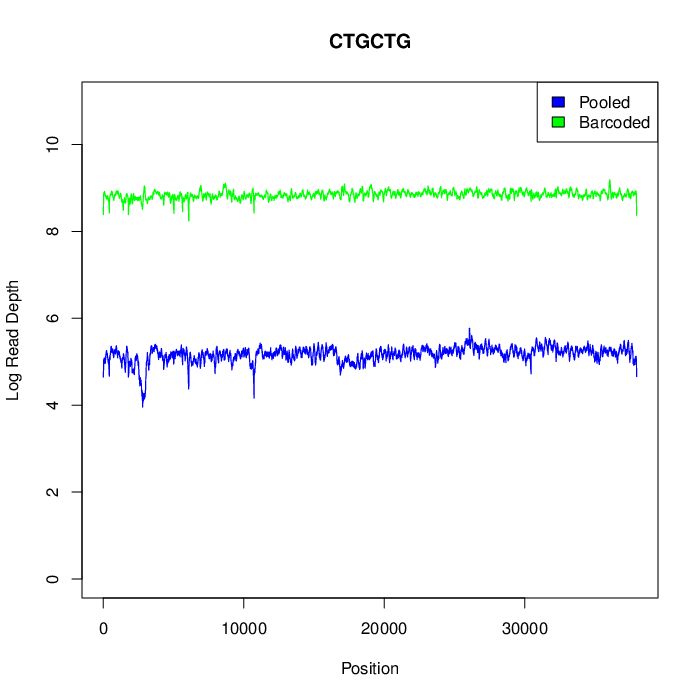

Supplement: File S1 — Estimated sequencing read depth across all clones. The read depth was plotted across each of the 73 clones for both barcoded and pooled sequencing. Read depth was estimated by comparing raw reads to the barcoded reference sequence. (ZIP) [file pone.0098968.s013.zip › CTGCTG.png]

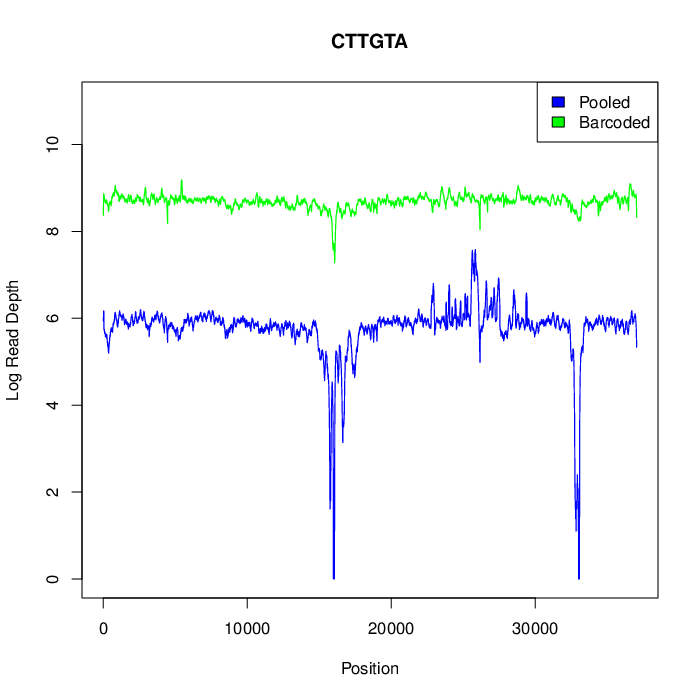

Supplement: File S1 — Estimated sequencing read depth across all clones. The read depth was plotted across each of the 73 clones for both barcoded and pooled sequencing. Read depth was estimated by comparing raw reads to the barcoded reference sequence. (ZIP) [file pone.0098968.s013.zip › CTTGTA.png]

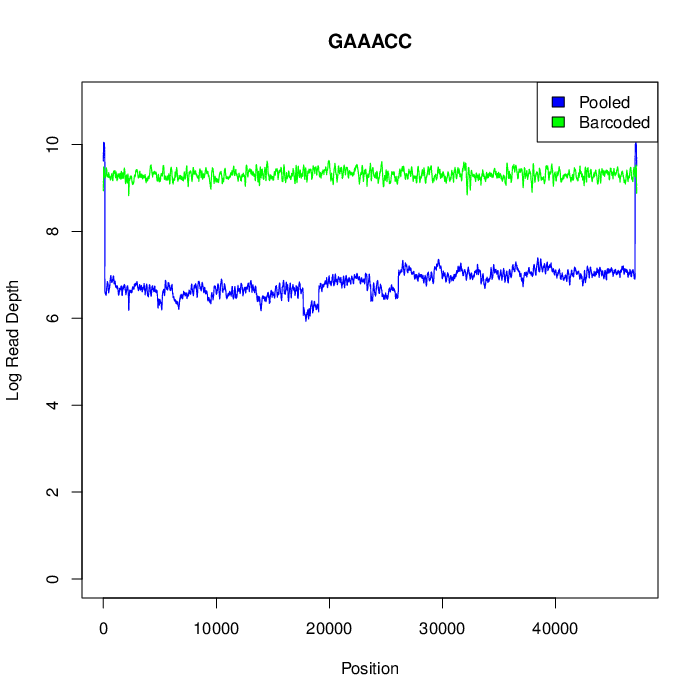

Supplement: File S1 — Estimated sequencing read depth across all clones. The read depth was plotted across each of the 73 clones for both barcoded and pooled sequencing. Read depth was estimated by comparing raw reads to the barcoded reference sequence. (ZIP) [file pone.0098968.s013.zip › GAAACC.png]

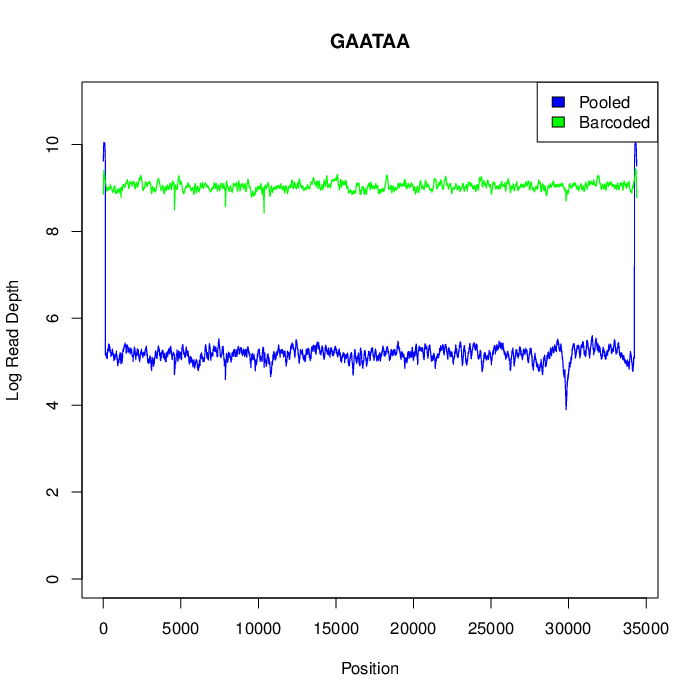

Supplement: File S1 — Estimated sequencing read depth across all clones. The read depth was plotted across each of the 73 clones for both barcoded and pooled sequencing. Read depth was estimated by comparing raw reads to the barcoded reference sequence. (ZIP) [file pone.0098968.s013.zip › GAATAA.png]

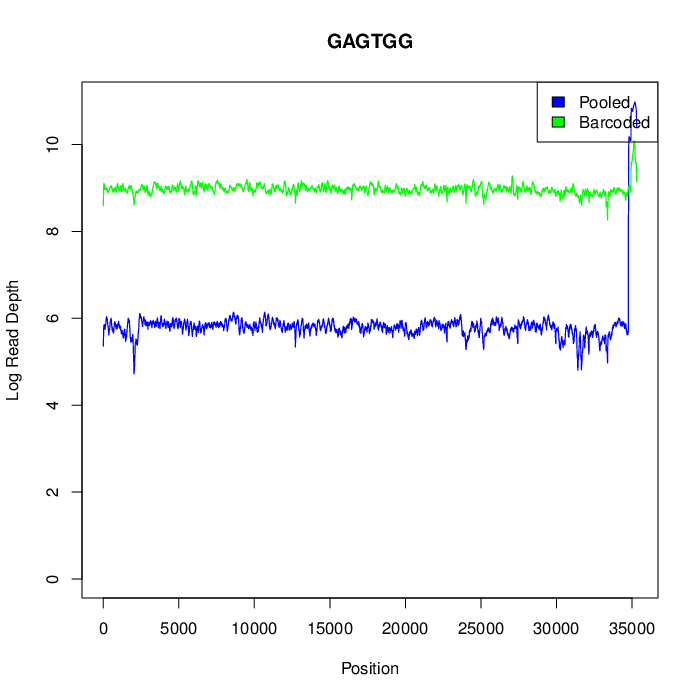

Supplement: File S1 — Estimated sequencing read depth across all clones. The read depth was plotted across each of the 73 clones for both barcoded and pooled sequencing. Read depth was estimated by comparing raw reads to the barcoded reference sequence. (ZIP) [file pone.0098968.s013.zip › GAGTGG.png]

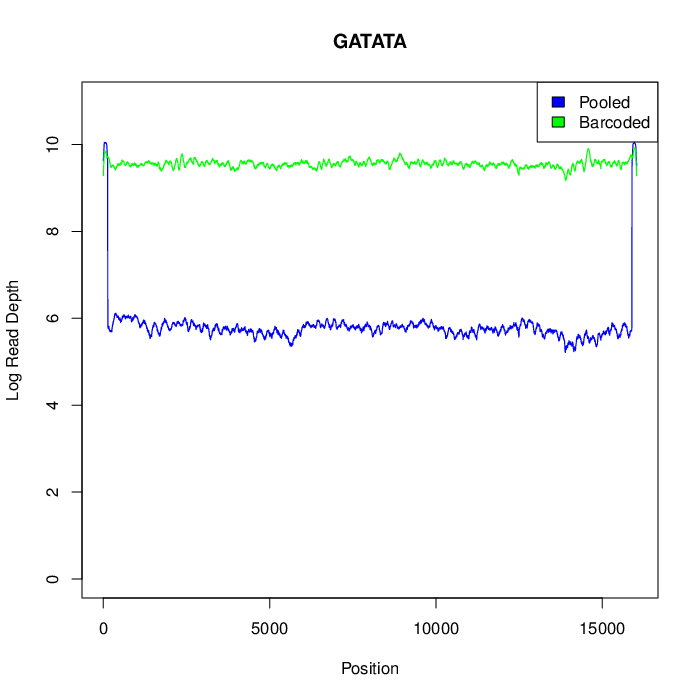

Supplement: File S1 — Estimated sequencing read depth across all clones. The read depth was plotted across each of the 73 clones for both barcoded and pooled sequencing. Read depth was estimated by comparing raw reads to the barcoded reference sequence. (ZIP) [file pone.0098968.s013.zip › GATATA.png]

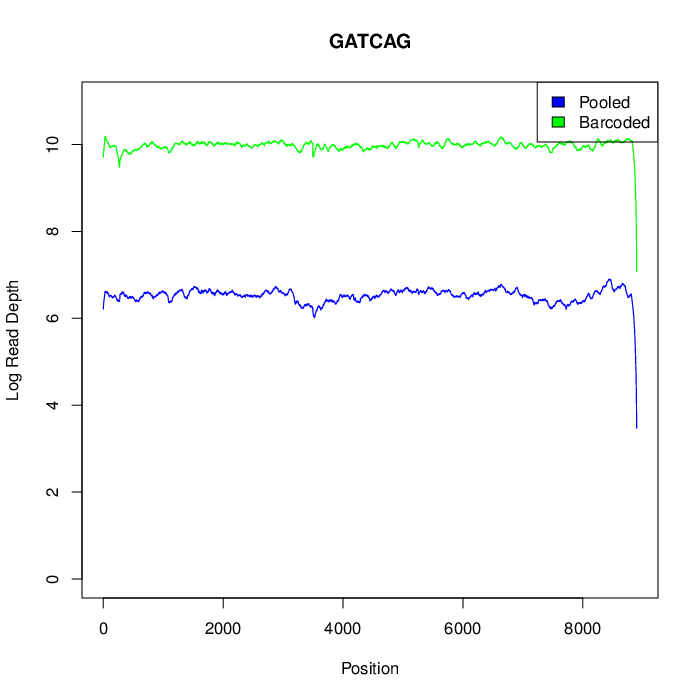

Supplement: File S1 — Estimated sequencing read depth across all clones. The read depth was plotted across each of the 73 clones for both barcoded and pooled sequencing. Read depth was estimated by comparing raw reads to the barcoded reference sequence. (ZIP) [file pone.0098968.s013.zip › GATCAG.png]

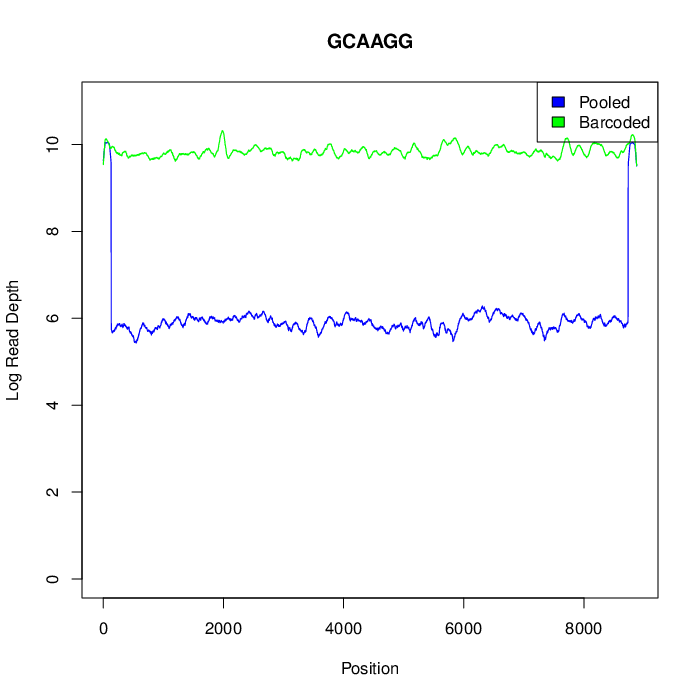

Supplement: File S1 — Estimated sequencing read depth across all clones. The read depth was plotted across each of the 73 clones for both barcoded and pooled sequencing. Read depth was estimated by comparing raw reads to the barcoded reference sequence. (ZIP) [file pone.0098968.s013.zip › GCAAGG.png]

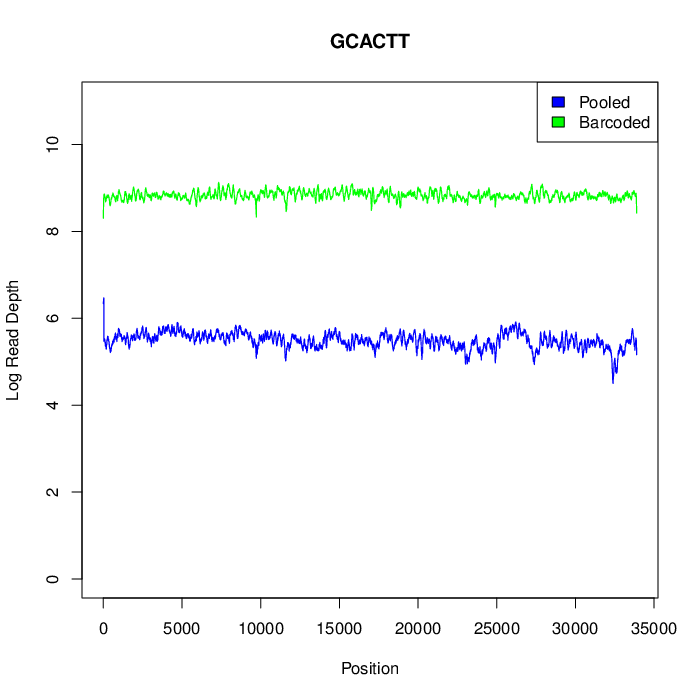

Supplement: File S1 — Estimated sequencing read depth across all clones. The read depth was plotted across each of the 73 clones for both barcoded and pooled sequencing. Read depth was estimated by comparing raw reads to the barcoded reference sequence. (ZIP) [file pone.0098968.s013.zip › GCACTT.png]

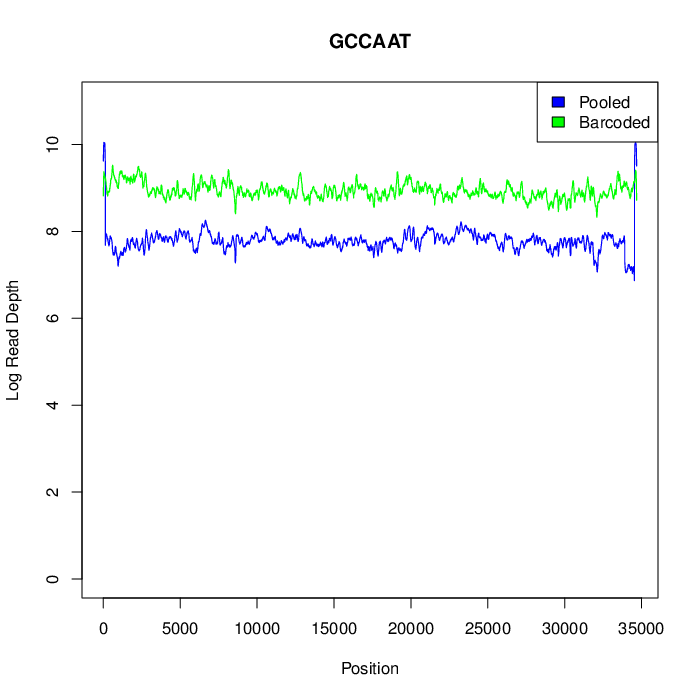

Supplement: File S1 — Estimated sequencing read depth across all clones. The read depth was plotted across each of the 73 clones for both barcoded and pooled sequencing. Read depth was estimated by comparing raw reads to the barcoded reference sequence. (ZIP) [file pone.0098968.s013.zip › GCCAAT.png]

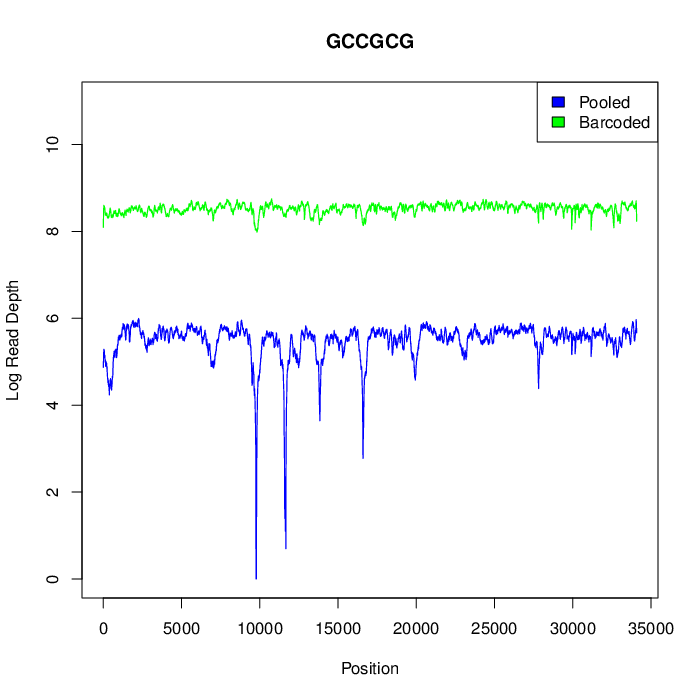

Supplement: File S1 — Estimated sequencing read depth across all clones. The read depth was plotted across each of the 73 clones for both barcoded and pooled sequencing. Read depth was estimated by comparing raw reads to the barcoded reference sequence. (ZIP) [file pone.0098968.s013.zip › GCCGCG.png]

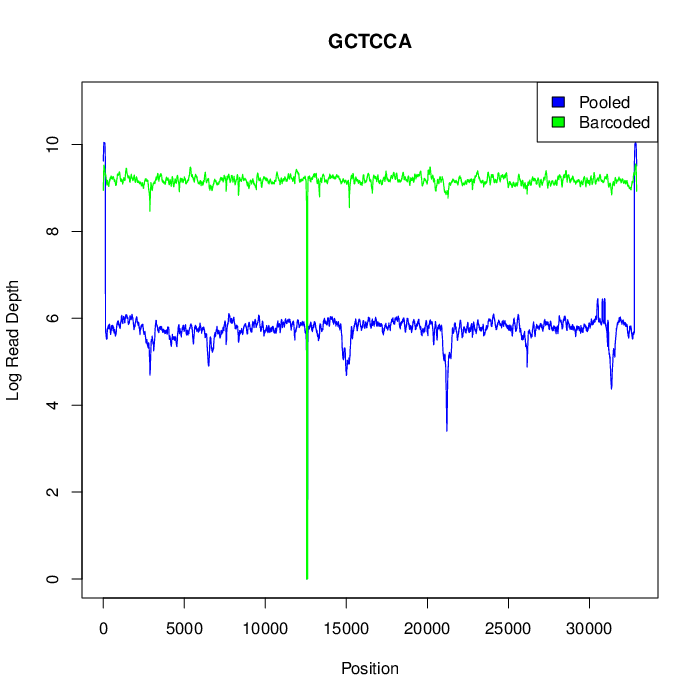

Supplement: File S1 — Estimated sequencing read depth across all clones. The read depth was plotted across each of the 73 clones for both barcoded and pooled sequencing. Read depth was estimated by comparing raw reads to the barcoded reference sequence. (ZIP) [file pone.0098968.s013.zip › GCTCCA.png]

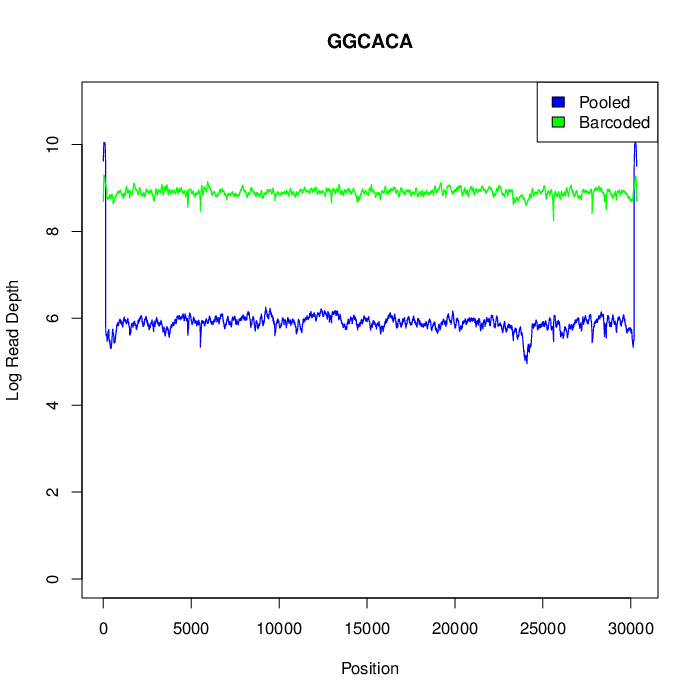

Supplement: File S1 — Estimated sequencing read depth across all clones. The read depth was plotted across each of the 73 clones for both barcoded and pooled sequencing. Read depth was estimated by comparing raw reads to the barcoded reference sequence. (ZIP) [file pone.0098968.s013.zip › GGCACA.png]

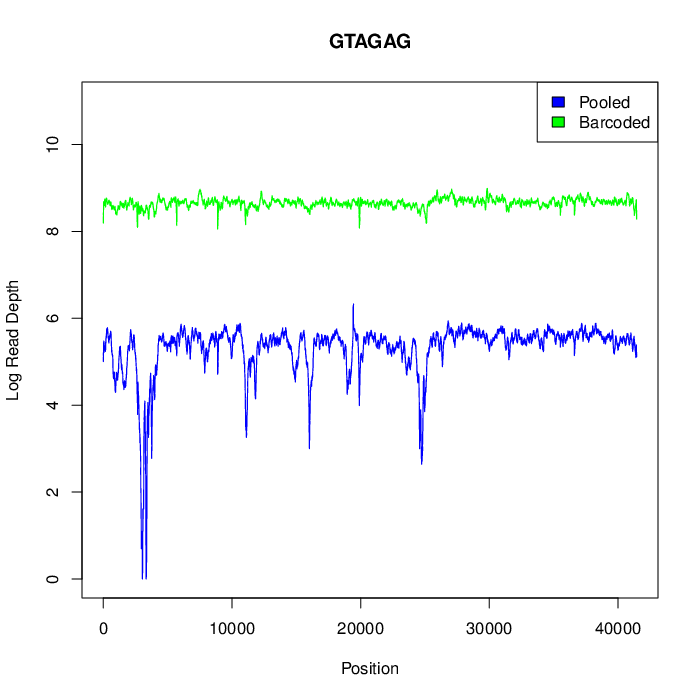

Supplement: File S1 — Estimated sequencing read depth across all clones. The read depth was plotted across each of the 73 clones for both barcoded and pooled sequencing. Read depth was estimated by comparing raw reads to the barcoded reference sequence. (ZIP) [file pone.0098968.s013.zip › GTAGAG.png]

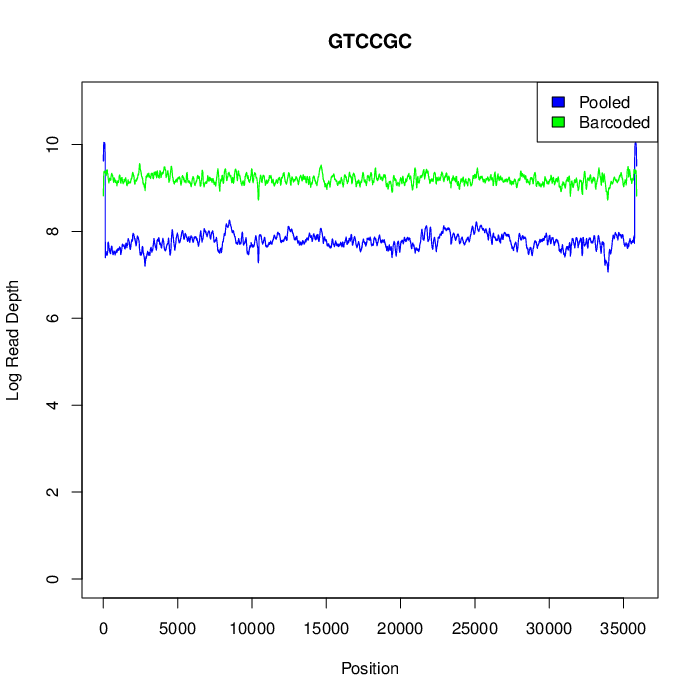

Supplement: File S1 — Estimated sequencing read depth across all clones. The read depth was plotted across each of the 73 clones for both barcoded and pooled sequencing. Read depth was estimated by comparing raw reads to the barcoded reference sequence. (ZIP) [file pone.0098968.s013.zip › GTCCGC.png]

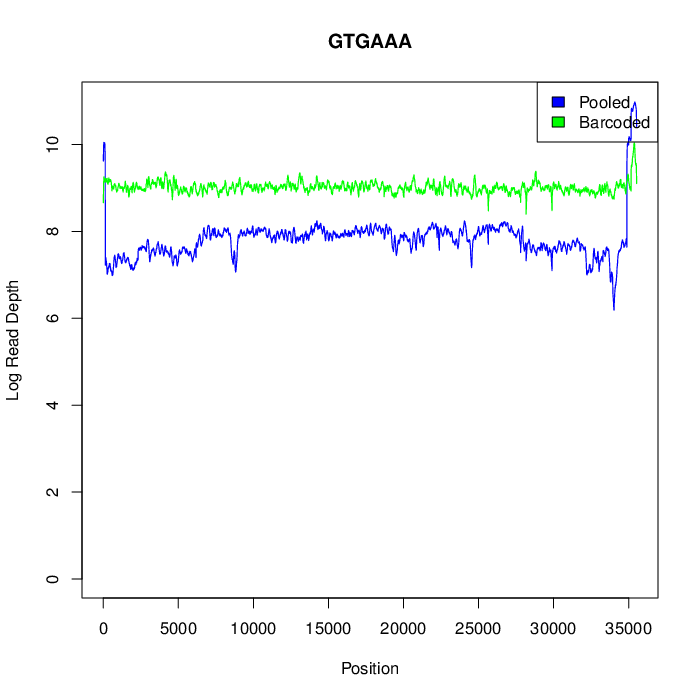

Supplement: File S1 — Estimated sequencing read depth across all clones. The read depth was plotted across each of the 73 clones for both barcoded and pooled sequencing. Read depth was estimated by comparing raw reads to the barcoded reference sequence. (ZIP) [file pone.0098968.s013.zip › GTGAAA.png]

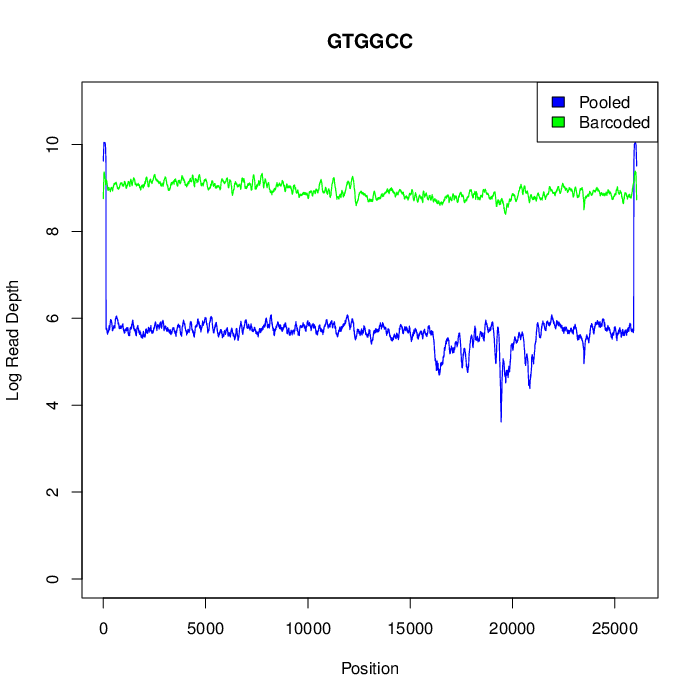

Supplement: File S1 — Estimated sequencing read depth across all clones. The read depth was plotted across each of the 73 clones for both barcoded and pooled sequencing. Read depth was estimated by comparing raw reads to the barcoded reference sequence. (ZIP) [file pone.0098968.s013.zip › GTGGCC.png]

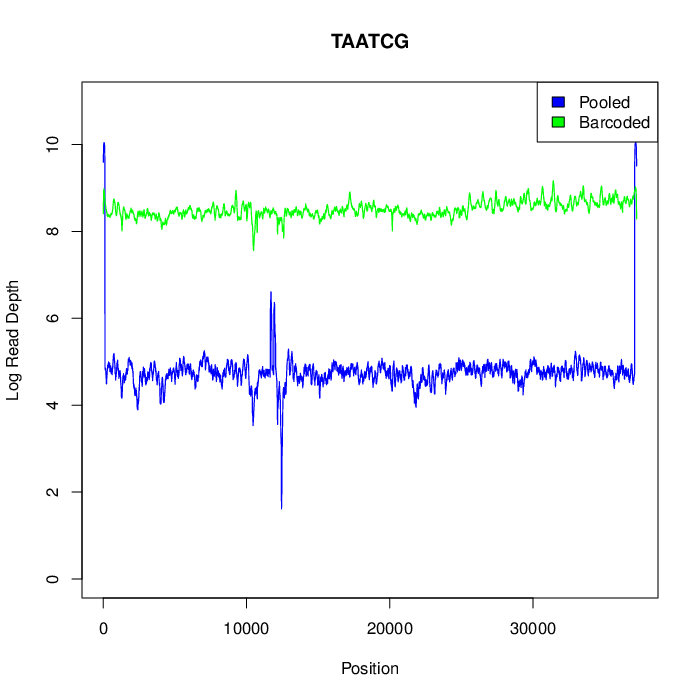

Supplement: File S1 — Estimated sequencing read depth across all clones. The read depth was plotted across each of the 73 clones for both barcoded and pooled sequencing. Read depth was estimated by comparing raw reads to the barcoded reference sequence. (ZIP) [file pone.0098968.s013.zip › TAATCG.png]

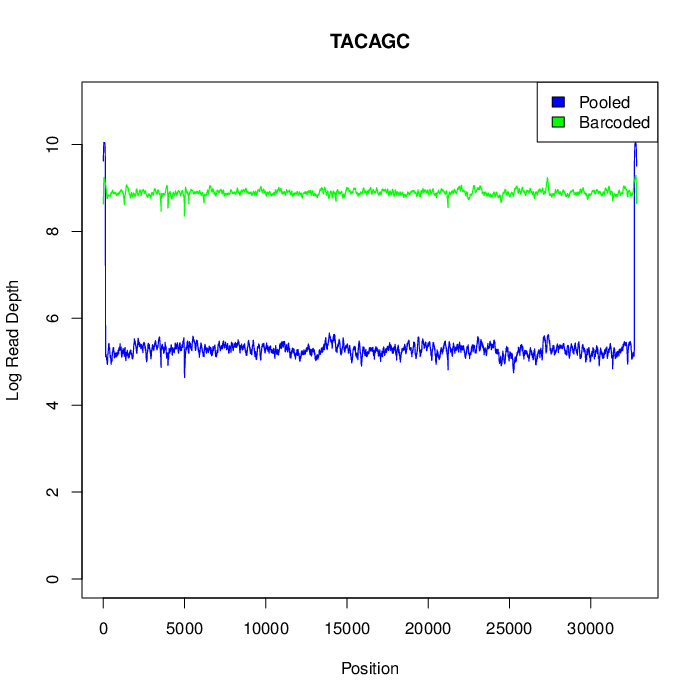

Supplement: File S1 — Estimated sequencing read depth across all clones. The read depth was plotted across each of the 73 clones for both barcoded and pooled sequencing. Read depth was estimated by comparing raw reads to the barcoded reference sequence. (ZIP) [file pone.0098968.s013.zip › TACAGC.png]

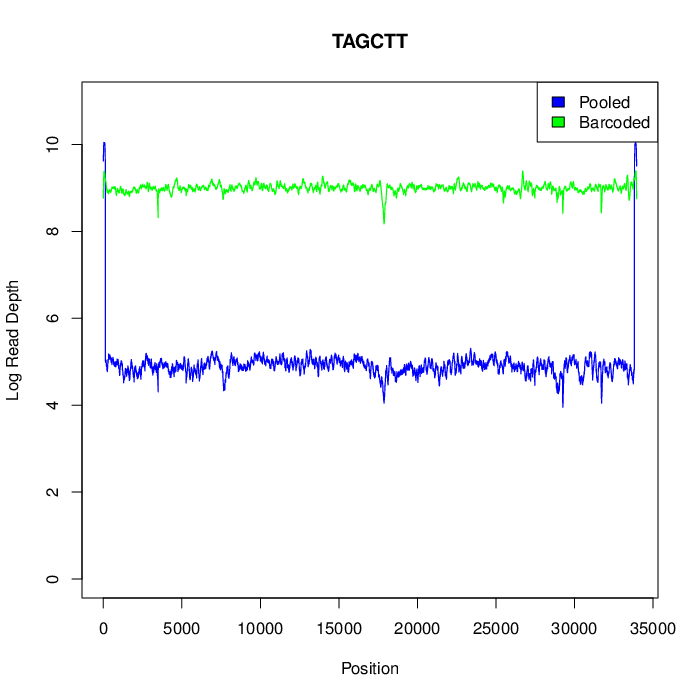

Supplement: File S1 — Estimated sequencing read depth across all clones. The read depth was plotted across each of the 73 clones for both barcoded and pooled sequencing. Read depth was estimated by comparing raw reads to the barcoded reference sequence. (ZIP) [file pone.0098968.s013.zip › TAGCTT.png]

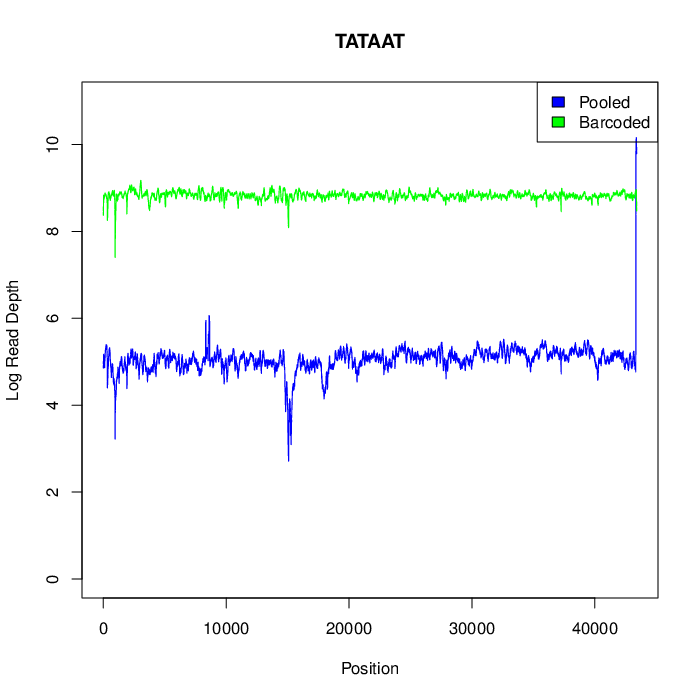

Supplement: File S1 — Estimated sequencing read depth across all clones. The read depth was plotted across each of the 73 clones for both barcoded and pooled sequencing. Read depth was estimated by comparing raw reads to the barcoded reference sequence. (ZIP) [file pone.0098968.s013.zip › TATAAT.png]

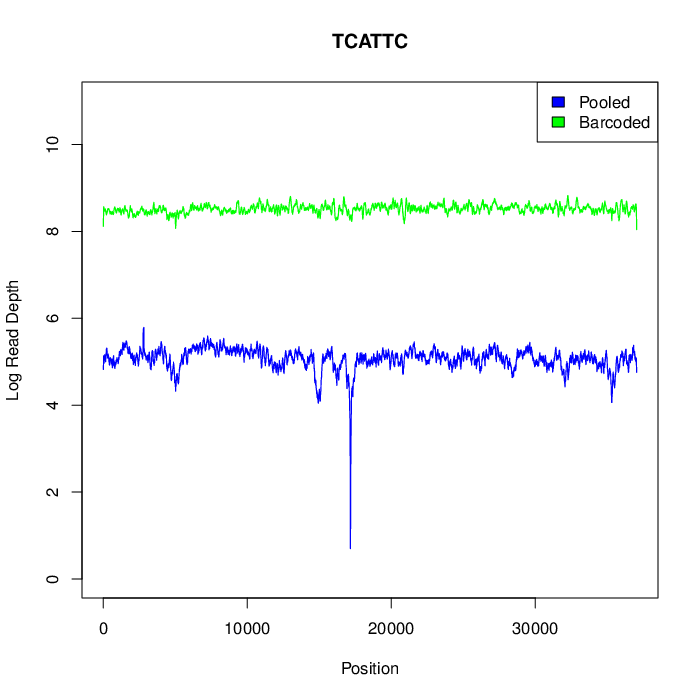

Supplement: File S1 — Estimated sequencing read depth across all clones. The read depth was plotted across each of the 73 clones for both barcoded and pooled sequencing. Read depth was estimated by comparing raw reads to the barcoded reference sequence. (ZIP) [file pone.0098968.s013.zip › TCATTC.png]

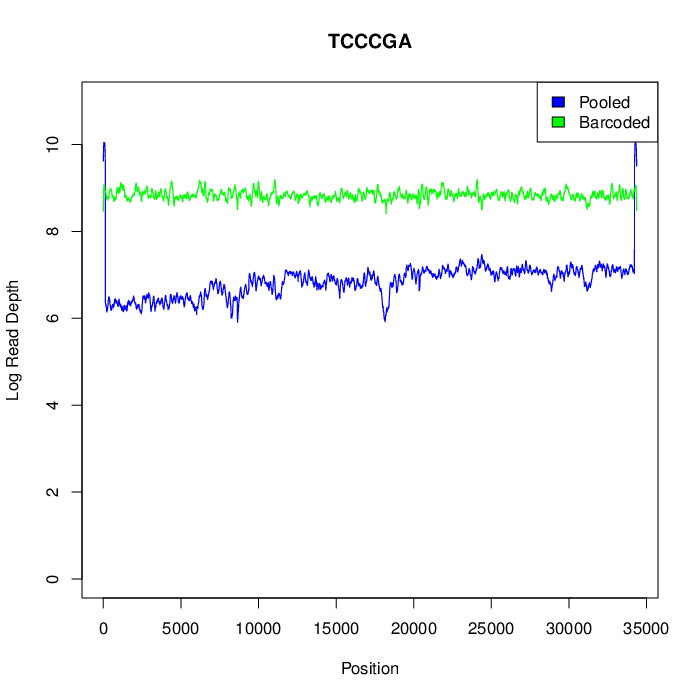

Supplement: File S1 — Estimated sequencing read depth across all clones. The read depth was plotted across each of the 73 clones for both barcoded and pooled sequencing. Read depth was estimated by comparing raw reads to the barcoded reference sequence. (ZIP) [file pone.0098968.s013.zip › TCCCGA.png]

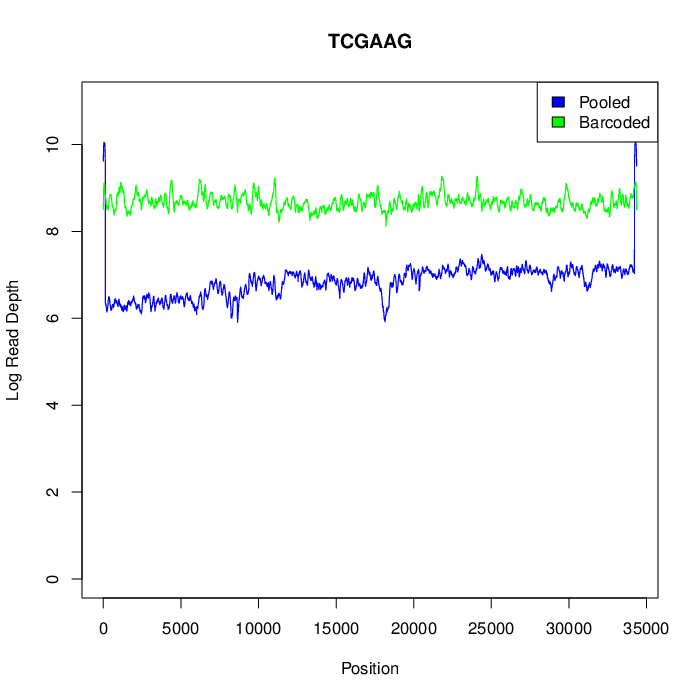

Supplement: File S1 — Estimated sequencing read depth across all clones. The read depth was plotted across each of the 73 clones for both barcoded and pooled sequencing. Read depth was estimated by comparing raw reads to the barcoded reference sequence. (ZIP) [file pone.0098968.s013.zip › TCGAAG.png]

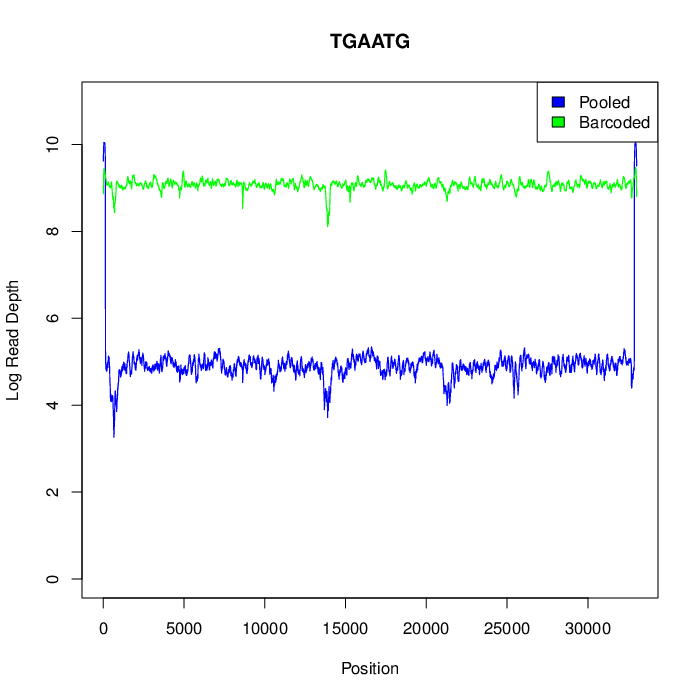

Supplement: File S1 — Estimated sequencing read depth across all clones. The read depth was plotted across each of the 73 clones for both barcoded and pooled sequencing. Read depth was estimated by comparing raw reads to the barcoded reference sequence. (ZIP) [file pone.0098968.s013.zip › TGAATG.png]

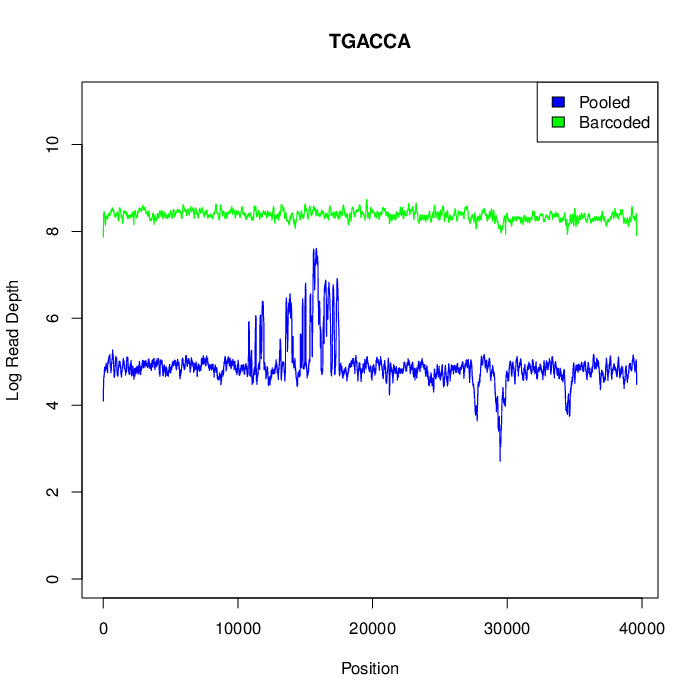

Supplement: File S1 — Estimated sequencing read depth across all clones. The read depth was plotted across each of the 73 clones for both barcoded and pooled sequencing. Read depth was estimated by comparing raw reads to the barcoded reference sequence. (ZIP) [file pone.0098968.s013.zip › TGACCA.png]

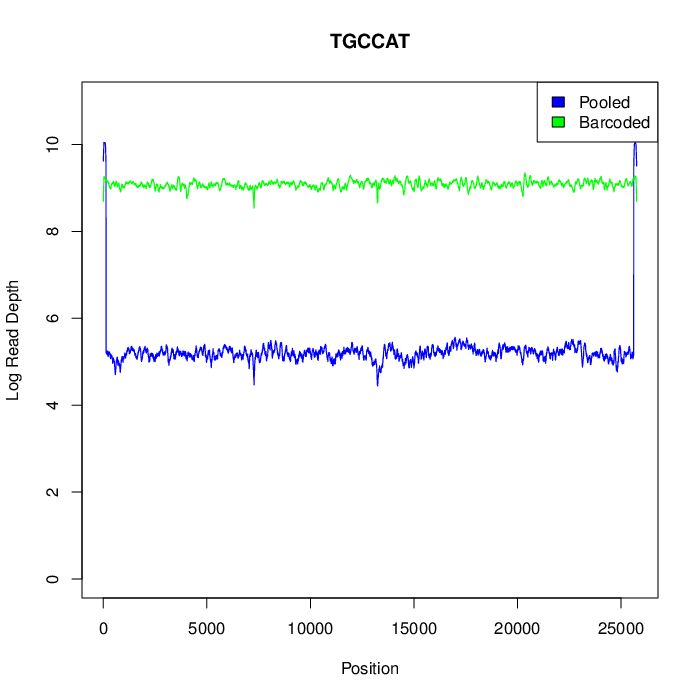

Supplement: File S1 — Estimated sequencing read depth across all clones. The read depth was plotted across each of the 73 clones for both barcoded and pooled sequencing. Read depth was estimated by comparing raw reads to the barcoded reference sequence. (ZIP) [file pone.0098968.s013.zip › TGCCAT.png]

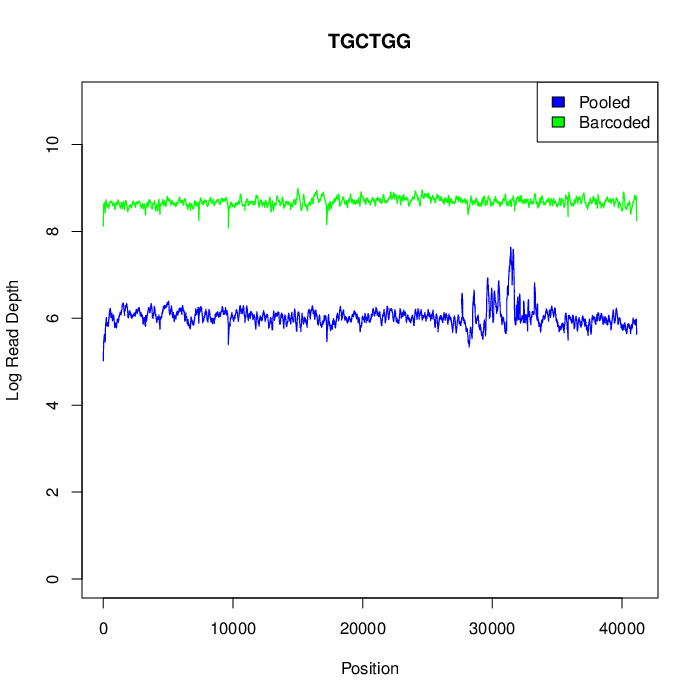

Supplement: File S1 — Estimated sequencing read depth across all clones. The read depth was plotted across each of the 73 clones for both barcoded and pooled sequencing. Read depth was estimated by comparing raw reads to the barcoded reference sequence. (ZIP) [file pone.0098968.s013.zip › TGCTGG.png]

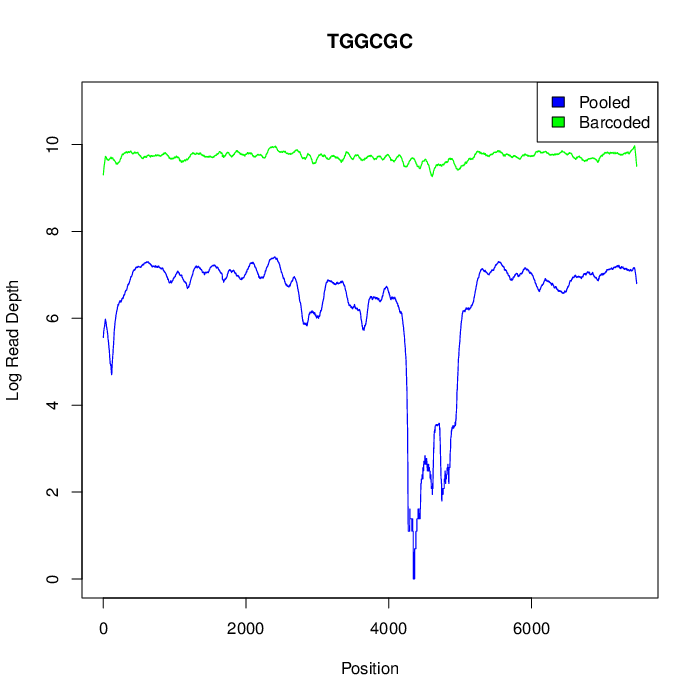

Supplement: File S1 — Estimated sequencing read depth across all clones. The read depth was plotted across each of the 73 clones for both barcoded and pooled sequencing. Read depth was estimated by comparing raw reads to the barcoded reference sequence. (ZIP) [file pone.0098968.s013.zip › TGGCGC.png]

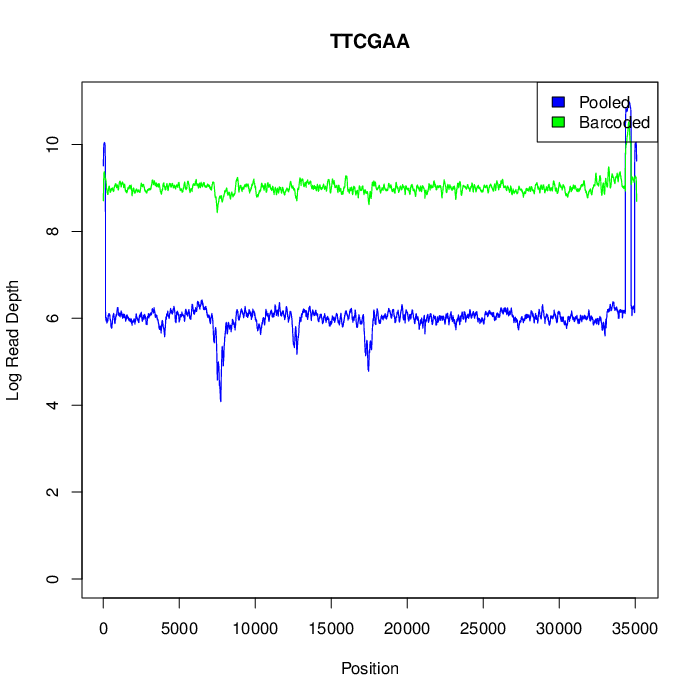

Supplement: File S1 — Estimated sequencing read depth across all clones. The read depth was plotted across each of the 73 clones for both barcoded and pooled sequencing. Read depth was estimated by comparing raw reads to the barcoded reference sequence. (ZIP) [file pone.0098968.s013.zip › TTCGAA.png]

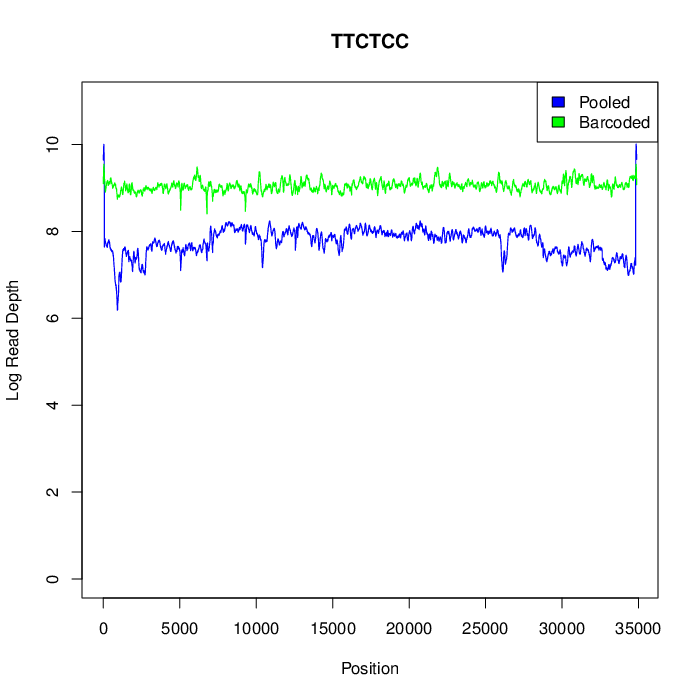

Supplement: File S1 — Estimated sequencing read depth across all clones. The read depth was plotted across each of the 73 clones for both barcoded and pooled sequencing. Read depth was estimated by comparing raw reads to the barcoded reference sequence. (ZIP) [file pone.0098968.s013.zip › TTCTCC.png]
